# Ultrafast laser-driven topological phase patterning

**DOI:** 10.1126/sciadv.aee8907

**Published:** 2026-09-18

**Authors:** Jianyu Wu, Arthur Niedermayr, Gaolong Cao, Oscar Grånäs, Jonas Weissenrieder

**Affiliations:** ^1^ Light and Matter Physics, School of Engineering Sciences, KTH Royal Institute of Technology, SE-100 44 Stockholm, Sweden.; ^2^ Materials Theory, Department of Physics and Astronomy, Uppsala University, Uppsala, Sweden.

## Abstract

Microscopic and dynamic control over quantum states is essential for bridging fundamental studies of material properties to device function. Realizing such control at combined high spatial resolution and ultrafast temporal precision remains a major challenge. Here, we demonstrate femtosecond laser-driven patterning of topological quantum states in the Weyl semimetal WTe_2_. By engineering the excitation field into a transient optical grating, we create spatially selective and reversible structural distortions that induce local transitions between topological and topologically trivial states. Using ultrafast transmission electron microscopy, we directly visualize the formation of a periodic Td/1T* heterostructure, observe the propagation of a phase front, and analyze nanoscale confinement of coherently excited optical phonon modes. Our findings establish a platform for all-optical, spatially programmable, and reconfigurable control of quantum states, paving the way for optically addressable topological devices.

## INTRODUCTION

Optical control of material properties with ultrashort laser pulses represents a rapidly evolving field of modern condensed matter physics (*1*–*5*). By providing access to dynamics on femtosecond to attosecond timescales, analysis using ultrashort laser pulses has unveiled a rich landscape of nonequilibrium phenomena from light-field–driven electron tunneling to transient superconductivity, with promise for development of future applications in quantum communication and ultrafast information processing (*6*, *7*).

A particularly fertile ground for such control is found in quantum materials, where the interplay between electronic, spin, and lattice degrees of freedom can give rise to previously unidentified transient states (*8*–*10*). A central challenge, however, lies in the spatially averaged nature of most ultrafast probes (*11*, *12*). Such probes obscures the spatial order—the microscopic arrangement of electronic and structural phases—that is fundamental to a material’s function and potential for device integration (*13*). Truly commanding the properties of these materials therefore requires ultrafast control with spatial specificity, targeting the relevant quantum degrees of freedom.

Structured optical fields present a powerful solution to this challenge. By sculpting light’s intensity, phase, and polarization at the wavelength scale, one can selectively and coherently excite specific electronic, phononic, or spin subsystems (*14*–*16*). This approach opens the door to creating and manipulating nonequilibrium quantum states with high spatial definition and ultrafast temporal precision.

In this work, we realize this potential by demonstrating ultrafast laser patterning of a topological quantum state. Using a transient optical grating (TOG) generated by the interference of two femtosecond pulses, we drive local reversible atomic displacements from the initial topological Td phase toward the topological trivial 1T* phase of WTe_2_. We directly visualize the resulting structural dynamics using ultrafast electron microscopy, achieving simultaneous nanometer and picosecond resolutions. This allows us to track the formation of a periodic Td/1T* heterostructure, observe the propagation of the phase front between the phases, and confirm the nanoscale confinement of a coherent optical phonon to the optically excited domains. Our findings establish a platform for noninvasive, spatially programmable, and reconfigurable control of quantum states, paving the way for optically addressable topological devices.

## RESULTS AND DISCUSSION

### Topological phase patterning

**Fig. 1.**
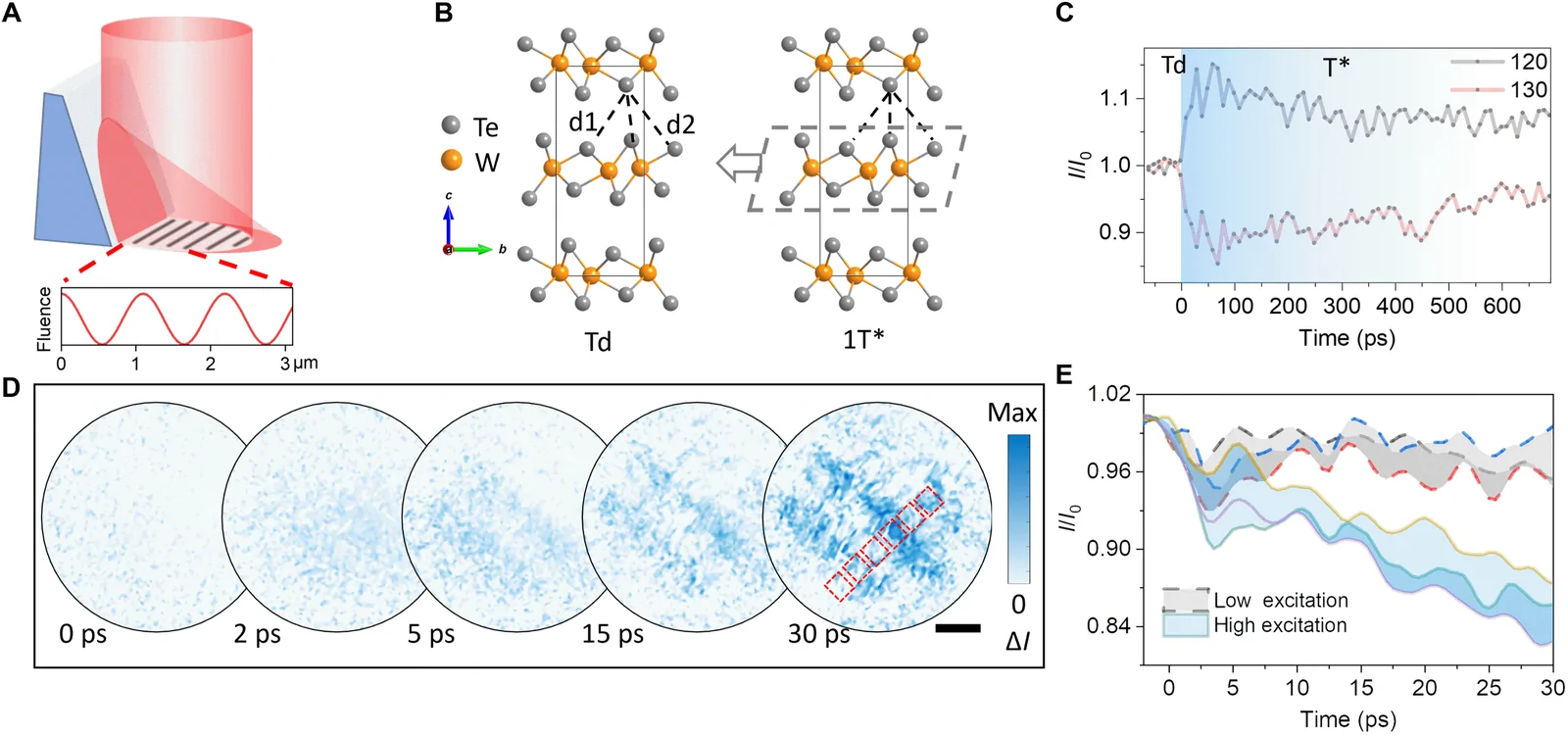
Light-driven structural phase patterning in WTe_2_. (**A**) Sketch of the TOG geometry formed by the interference between a direct laser beam and a beam reflected from a mirror placed in proximity to the sample. The pump beam is a TOG with a fluence ranging from 0.4 to 13.7 mJ/cm^2^ at a periodicity of 1.1 μm. (**B**) Atomic structure models of the topological Td-WTe_2_ phase with bond length d1 < d2 and the topologically trivial 1T* WTe_2_ phase with d1 = d2 (*9*). (**C**) Time traces of the diffraction intensities of Bragg spots (120) and (130) during the structural transition between the Td and 1T* phases. (**D**) DF difference images at increasing time delay collected from the (130) spot from a WTe_2_ sample excited by a TOG. A reference image, collected at −2 ps, is subtracted from each frame. Scale bar, 1 μm. (**E**) Time-dependent intensity traces from the six regions of interest (ROIs) indicated in the last frame in (D). Three of the ROIs are from spatial coordinates undergoing a phase transition (high local fluence), whereas the other three are from regions where the transition does not take place (low local fluence).

At above threshold excitation, the ground-state Td-WTe_2_ experiences a collective structural distortion with atomic coordinates approaching the metastable 1T* state (*9*, *17*). This structural distortion is induced by relative shear displacements between neighboring layers along the crystallographic *b* axis (*9*, *17*), which drives the lattice toward a centrosymmetric structure (Fig. 1B), leading to annihilation of the Weyl points already before reaching the 1T* coordinates (*9*, *18*, *19*). Thus, manipulation of the structural phase of WTe_2_ by femtosecond laser provides a path for tuning topological invariants at high spatiotemporal resolutions. We note that the present experiments probe structural degrees of freedom only; direct characterization of the electronic properties and topological invariants would require complementary electronic probes. In diffraction experiments, the shear displacement manifests as alternating intensity changes in the Bragg spots aligned with the *b* axis. Specifically, the (120) and (130) reflections show opposite intensity evolutions with increasing shift in *b*. Thus, these diffraction spots provide sensitive indicators for the transition (Fig. 1C). The reorganization of the atomic structure is completed within the first tens of picoseconds after laser excitation and the excited state persists for hundreds of picoseconds before it recovers to the Td ground state.

The interlayer shear displacement of WTe_2_ increases linearly with pump fluence above a threshold fluence (*17*), which opens the possibility of manipulating the local structural phase and topology through spatially varying the laser fluence. We engineer the optical excitation into a TOG by splitting and recombining a femtosecond laser pulse at the sample plane. This results in a sinusoidal fluence modulation with a periodicity in our geometry of ∼1.1 μm (Fig. 1A). The TOG serves as a spatial template, imprinting alternating high- and low-fluence regions across the sample surface. Calculations for local pump fluence and periodicity are provided in fig. S1.

The structural phase transition process in WTe_2_ with TOG excitation is studied by dark-field (DF) imaging. The DF images collected from the 130 Bragg spot show the formation of spatially patterned structural phases (Fig. 1D). To enhance the dynamic image contrast, we subtract a reference image collected at −2 ps (fig. S2) from all time delays. Dark fringes appear from ∼2 ps time delay and become increasingly intense until 30 ps. The decrease in local Bragg intensity at the fringes indicates that the local lattice structure changes from Td toward 1T*. The fringes are fully established at 30 ps, with orientation and spatial periodicity in agreement with those of the TOG. Intensity traces from multiple regions of interest (ROIs) highlight the phase separated response (Fig. 1E): ROIs at spatial sample positions excited with high local fluence exhibit a pronounced intensity drop (∼20% at 30 ps), indicative of a local phase transition to the 1T* phase. The diffraction intensity from regions in between remains almost unperturbed. Superimposed on these transitions are coherent oscillations attributed to the optical A_1_ shear phonon mode, which further reflect the lattice dynamics underlying the phase transition. These results demonstrate a powerful concept: By using spatially structuring light, we can imprint nanoscale patterns of topological and trivial phases into a quantum material and track their evolution in real time.

### Phase propagation

**Fig. 2.**
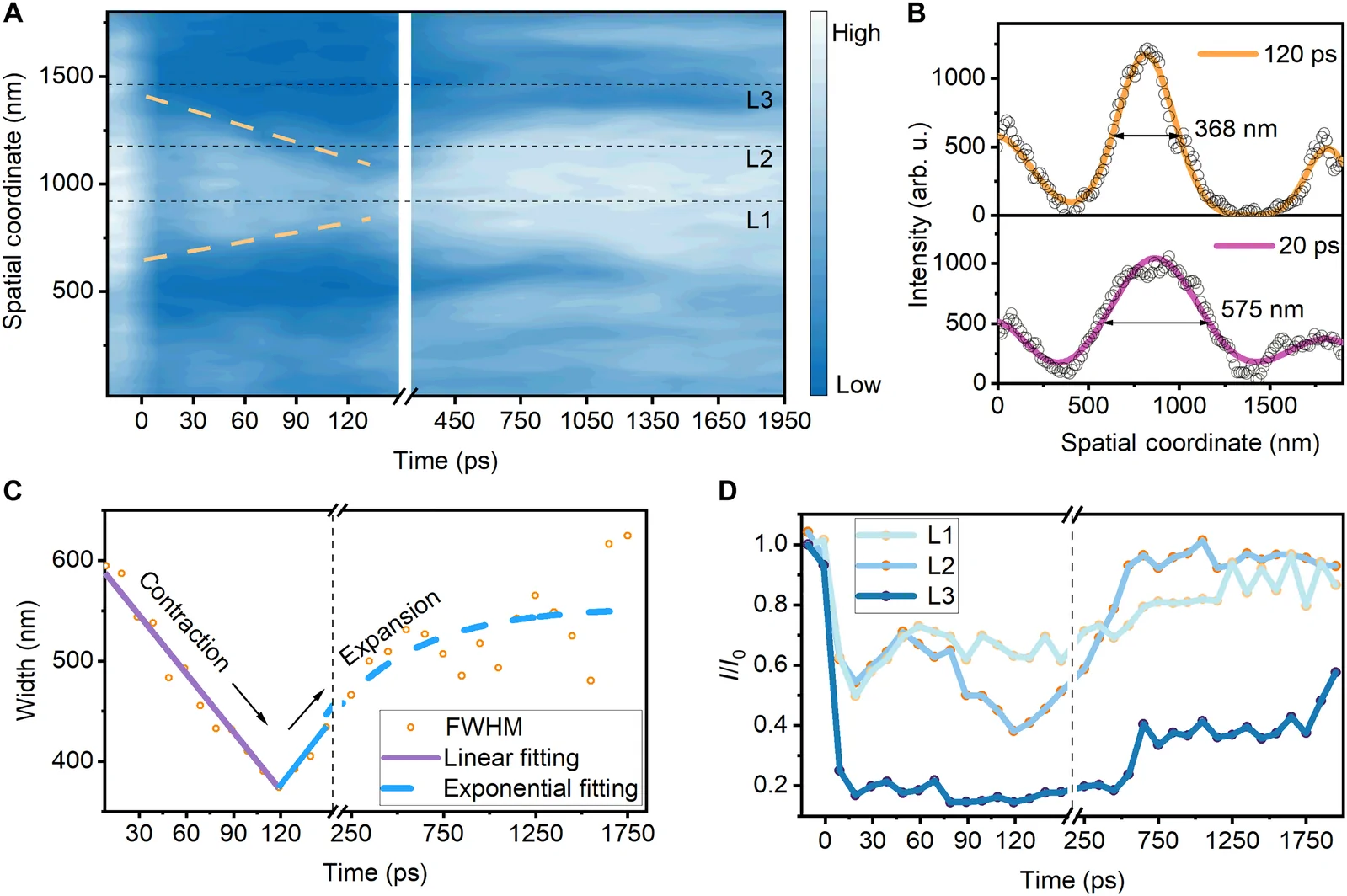
Phase evolution in space and time domains. The pump beam is a TOG with a fluence ranging from 0.1 to 3.5 mJ/cm^2^ at a periodicity of 1.1 μm. (**A**) STCP generated from a DF image series using the (150) diffraction spot of WTe_2_ excited with a TOG. Note: time steps are different before and after 150 ps. Two yellow dashed lines highlight interfaces between different domains. (**B**) Column intensity for the 20- and 120-ps time delays in the STCP. The solid lines represent Gaussian fits, with FWHM indicated for the central peak. (**C**) Extracted FWHM as a function of time delay. The results before and after 120 ps are fitted with two straight lines. The results after 150 ps are fitted with an exponential function. (**D**) Intensity profiles extracted from the black dashed horizontal lines in (A).

An understanding of the mechanisms governing phase separation and interaction between phases is critical for the local manipulation of topological invariants. Through DF imaging, we monitor the evolution of adjacent Td and 1T* phase domains and the position of the interfaces during the first 2 ns following laser excitation (movie S1). Sample regions subjected to local high fluence exhibit an abrupt drop in intensity within 20 ps (300 to 700 nm and 1300 to 1700 nm, Fig. 2A), indicative of transition toward the 1T* phase. In contrast, low-fluence regions retain near-constant intensity, indicative of these regions remaining in the Td phase. Sharp boundaries form at the interface between these distinct responses. These boundaries are here defined as the phase fronts. The DF contrast provides a direct visualization of the propagation of the phase fronts.

Dark domains, corresponding to the 1T* phase, expand laterally around local fluence maxima, reaching maximum extension at ∼120 ps before receding and eventually disappearing by ∼2 ns. This temporal behavior reflects the excitation, growth, and relaxation of the excited state. A three-term Gaussian model was used to fit the column intensity for all time delays and extract the full width at half maximum (FWHM) of the central Td phase domain in the space time contour plot (STCP). Figure 2B shows extracted line profiles at 20 and 120 ps, where the decrease in domain size with time is evident. Notably, the phase front propagates at a nearly constant velocity of ∼1.0 nm/ps, as extracted from linear fits to domain widths at successive delays (Fig. 2C). The extracted velocity is comparable to the shear-wave velocity in WTe_2_ (∼1.6 nm/ps) (*20*), suggesting that the phase propagation is strain driven rather than thermally mediated, as thermal diffusion would be orders of magnitude slower (fig. S5).

Temporal DF analyses of locations within the TOG experiencing different local fluences provide insights into how the propagation of the phase front governs the dynamics of the local atomic structure. The horizontal dashed lines in Fig. 2A indicates three representative positions within the TOG: L1, located at the center of a Td phase domain (minimum local fluence, ∼0.1 mJ/cm^2^); L2, situated at the interface between the 1T* and Td phases (intermediate local fluence, ∼1.8 mJ/cm^2^); and L3, positioned at the center of a 1T*-like domain (maximum local fluence, ∼3.5 mJ/cm^2^). At L3, the intensity rapidly decreases to ∼20% of its pre-excitation value (Fig. 2D). The change in diffraction intensity correlates with the magnitude of the interlayer shear displacement, allowing displacement estimates via structural factor calculations (*17*). The shear displacement at L3 is approximately 23 pm along the *b* direction. This displacement is notably larger than what has been reported for homogeneous excitation and approaches the 37 pm required to complete the 1T* structural transition (*9*), reflecting the higher local excitation fluence achievable when energy is confined to nanometer-scale regions (*17*), effectively mitigating thermal damage by minimizing heat accumulation. We note that the estimated displacement of ∼23 pm is averaged over the full 34-nm sample thickness. The local displacement within the optically excited near-surface region is therefore expected to be considerably larger. At the L2 position, the system experiences a two-step transition. The initial lattice distortion is insufficient to fully overcome the potential energy barrier and reach the 1T* local minimum. Because the system does not cross this barrier, a partial recovery toward the Td phase begins already before 30 ps. This recovery is subsequently interrupted and reversed at around 50 ps, leading to a secondary decrease in intensity that reaches a minimum at approximately 120 ps. This timescale coincides with the arrival of strain waves emanating from neighboring 1T* domains. In contrast, L1 exhibits only modest transient lattice distortions due to the low local excitation fluence. After 750 ps, L3 remains in a distorted structural state (interlayer displacement ∼16 pm) corresponding to a topologically trivial phase, whereas L1 and L2 have recovered to the Td ground state. This spatially nonuniform recovery establishes a transient Td/1T* structural heterostructure, creating regions associated with distinct topological electronic properties.

Together, these observations establish that the metastable 1T* state propagates through lattice-mediated strain. Local laser excitation induces lattice distortions that generate a nonlocal release of stress into neighboring regions, thereby driving the propagation of the phase front. The comparable velocities of the phase front and the shear wave further support this interpretation. This mechanism illustrates how a spatially localized optical excitation can trigger collective structural rearrangements over micrometer-scale distances, providing a pathway for nonlocal phase engineering.

### Spatial and frequency mapping of optical phonons

**Fig. 3.**
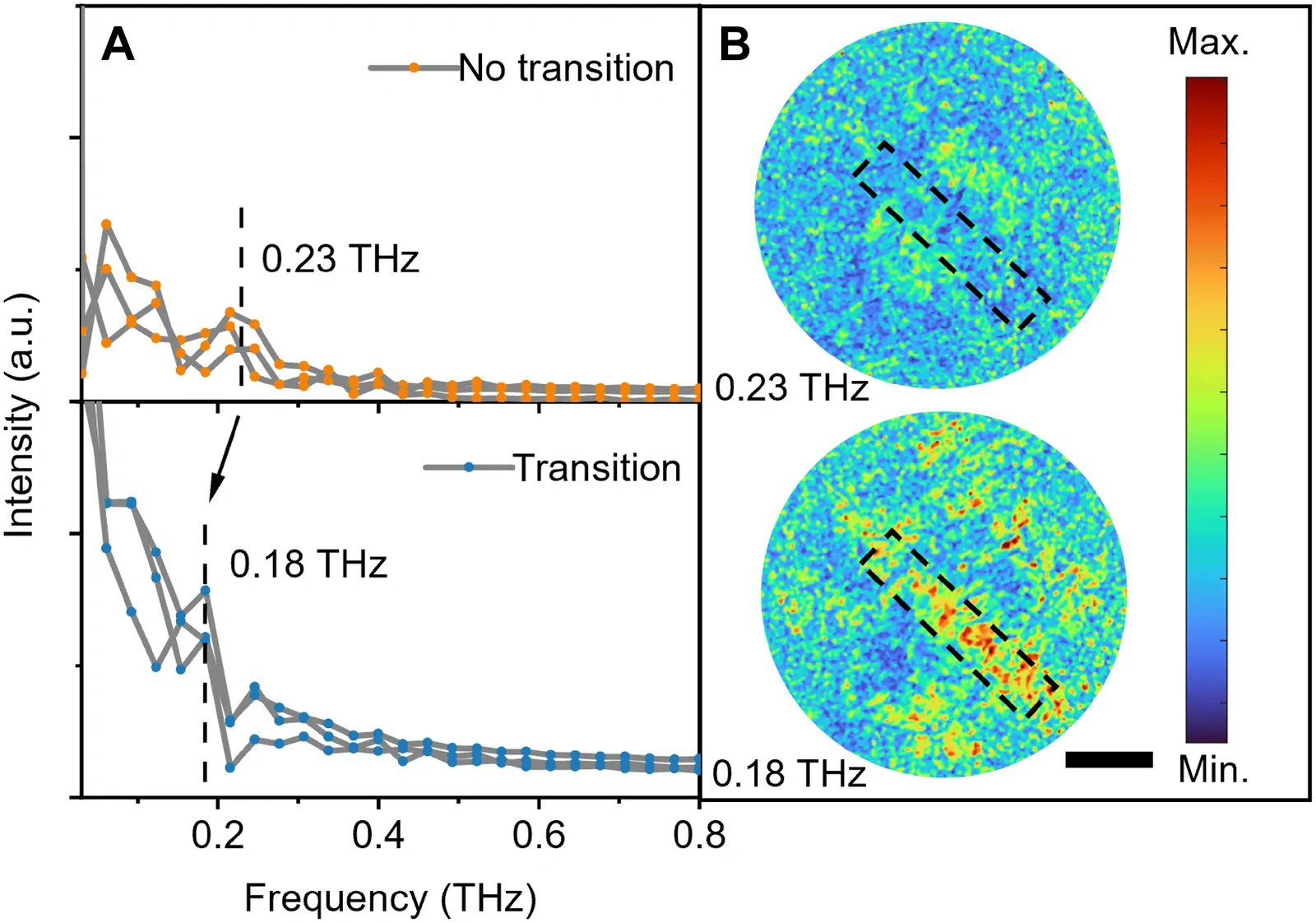
Mapping the excitation of optical phonons. (**A**) A_1_ mode phonon softening with frequency indicated. The results are obtained by performing FFT on the data traces in Fig. 1E. (**B**) FFT amplitude mapping at two specific frequencies, 0.23 and 0.18 THz. The dashed rectangles highlight a region exhibiting phonon softening. The spatial FFT mapping of the A_1_ shear mode is constructed from the DF image series in Fig. 1. Each pixel represents the FFT amplitude ratio between the selected frequency and sum of total frequencies. Scale bar, 1 μm.

Femtosecond optical excitation of WTe_2_ not only drives structural transitions but also excites coherent interlayer shear phonons (A_1_) that induce a lattice distortion analogous to that involved in the Td-to-1T* transition. The A_1_ mode modulates the relative displacement of adjacent layers along the *b* axis (*17*). This periodic lattice displacement generates out-of-phase intensity oscillations of the (120) and (130) diffraction spots at a frequency close to 0.23 THz (*9*). To spatially resolve the TOG excited phonon mode, we performed nano-beam diffraction to probe structural oscillations at positions of different excitation fluence (fig. S10). The oscillating frequency exhibits a clear spatial dependence (fig. S11), confirming that the A_1_ mode is modulated by the structured optical field. Notably, we observe a distinct phonon softening at a spatial periodicity matching that of the optically induced phase patterning. In regions experiencing the Td-to-1T* transition, the A_1_ phonon red shifts from 0.23 THz to approximately 0.18 THz (Fig. 3A). These regions share the same periodicity and orientation as the TOG (Fig. 3B) and display a complementary spatial distribution to the 0.23-THz regions, reflecting the fluence sensitivity of the excitation. Such frequency softening indicates a reduction in the interlayer forces as the lattice approaches the 1T* phase. In addition to structural effects, local lattice heating may also contribute to the observed frequency shift (*21*, *22*). These results demonstrate that optical fields can be engineered to confine coherent lattice vibrations to submicrometer domains.

### Multidimensional mapping of patterned states by U-STEM

**Fig. 4.**
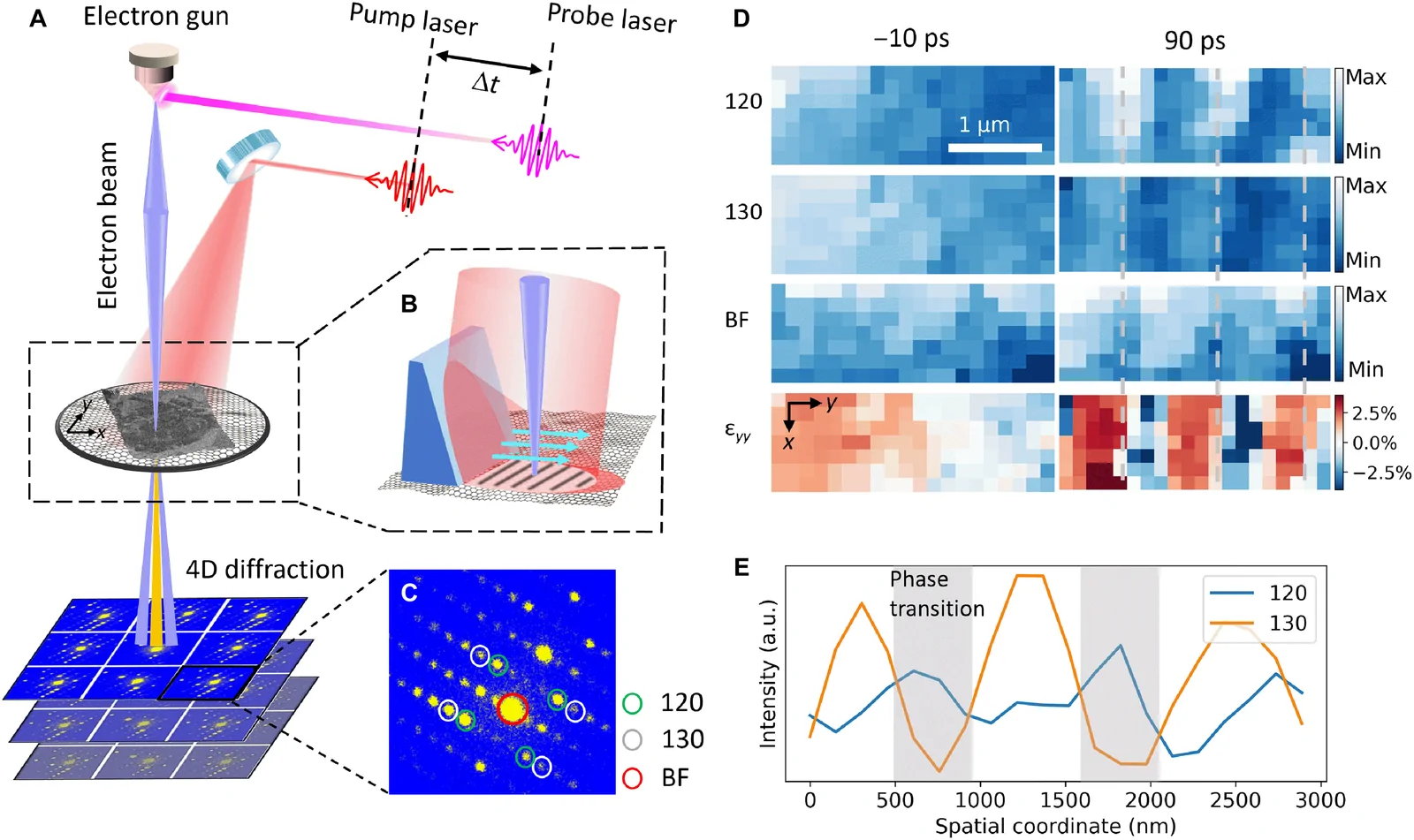
U-STEM analysis of local structural phase composition and strain. (**A**) Illustration of the U-STEM setup. Laser pulses are directed at the TEM cathode to generate electron pulses (probe). The probe position at the sample is controlled by the instrument scanning coils. A coupled laser pulse is directed to the sample (pump). A detector collects diffracted electrons as the probe scans the sample at different spatial and temporal coordinates. (**B**) Enlarged view of the formation of the TOG pump and the scanning path of the probe. The pump fluence range from 0.1 to 3.5 mJ/cm^2^ at a periodicity of 1.1 μm. (**C**) An electron diffraction pattern collected from WTe_2_. Equivalent Bragg spots are summed up to construct virtual bright- or dark-field images. (**D**) Images constructed from diffraction spots (120), (130), transmitted beam (BF), and strain maps at −10- and 90-ps time delay, after a 2 × 2 median filter. Dashed lines indicate the position of local sample bending (in BF). (**E**) Intensity sum plot (smoothed) of DF after removal of the contribution from local structural bending.

Unambiguous identification of local structural transitions requires excluding contributions from other possible lattice distortions, such as photoexcited coherent acoustic phonons. This requirement necessitates to move beyond the single–lattice plane information accessible in conventional DF or bright-field (BF) imaging. Four-dimensional scanning transmission electron microscopy (4D-STEM) overcomes this limitation by recording diffraction patterns from multiple lattice planes at each probe position, thereby capturing a comprehensive description of local crystallography and strain fields (*23*). Here, we extend 4D-STEM into the time domain by integrating femtosecond optical excitation with stroboscopic electron probing, realizing picosecond-resolved ultrafast 4D-STEM (U-STEM, Fig. 4A) of the phase-patterned WTe_2_ system. A ∼200-nm electron probe was rastered across the sample, recording diffraction patterns at each spatial coordinate and pump-probe delay. DF and BF images can be reconstructed by integrating intensity from selected Bragg reflections, allowing visualization of structural states from multiple lattice planes (Fig. 4D). These reconstructed images exhibit contrast fringes whose periodicity and orientation match the exciting TOG, but with slight position shift between frames, arising from the combined effects of local structural bending of the sample and change in atomic structure. The contrast in the BF channel reflects periodic structural sample bending, similar to previous observations of acoustic phonon–driven deformation (*24*), while the DF images reveal the superimposed phase-transition pattern. The local phase distribution can be isolated by subtraction of the bending contribution extracted from the BF signal (fig. S8). The validity of this method is evidenced by the antiphase intensity distribution between 120 and 130 (Fig. 4E). In addition, U-STEM provides direct access to time-resolved strain fields by tracking reciprocal-space shifts of Bragg peaks. The ε*_yy_* strain component displays a striped profile aligned with the optical excitation. Because the lattice parameter remains constant during the Td → 1T* transition, the detected strain arises predominantly from photoinduced sample bending. These results demonstrate the capability of U-STEM to simultaneously probe multiple diffraction channels, providing a multidimensional view of excited-state dynamics. A single line scan dataset resolves both the phase patterning and coherent optical phonons (fig. S7). Such rich diffraction dataset allows quantitative mapping of structure, crystal orientation, strain, and even electric and magnetic fields across space and time (*25*, *26*), enabling U-STEM as a versatile platform for probing ultrafast, spatially resolved phenomena with nanometer spatial and picosecond temporal resolution.

### Estimating topological robustness in finite domains

The topological classification is rooted in global properties of the electronic band structure. A key question for optically patterned WTe_2_ is whether finite Td domains embedded in a 1T* matrix preserves their topological character. In Weyl semimetals, finite-size confinement can cause surface states from opposite sides to overlap and hybridize, opening gaps that eliminate Weyl crossings. In the case of a type II Weyl semimetal, we must ensure that finite-size effects do not remove protected band crossings. Robust topology therefore remains well defined when the domain size is much larger than the decay length of localized Wannier states.

We investigate this by parameterizing a tight-binding model, constructing finite slabs, and investigating the hybridization gap Δ*E*. Further, following the framework developed by Benito-Matías and Molina (*27*) for finite-sized slab geometries, we derive a continuum expression for the hybridization gap that is used to estimate the hybridization gap for in-plane domains (see the Supplementary Materials for details). The parameters of the model ξ are estimated by fitting to the parameterized tight-binding model for finite geometries. From an experimental perspective, angular resolved photoemission may also be used (*28*).

**Fig. 5.**
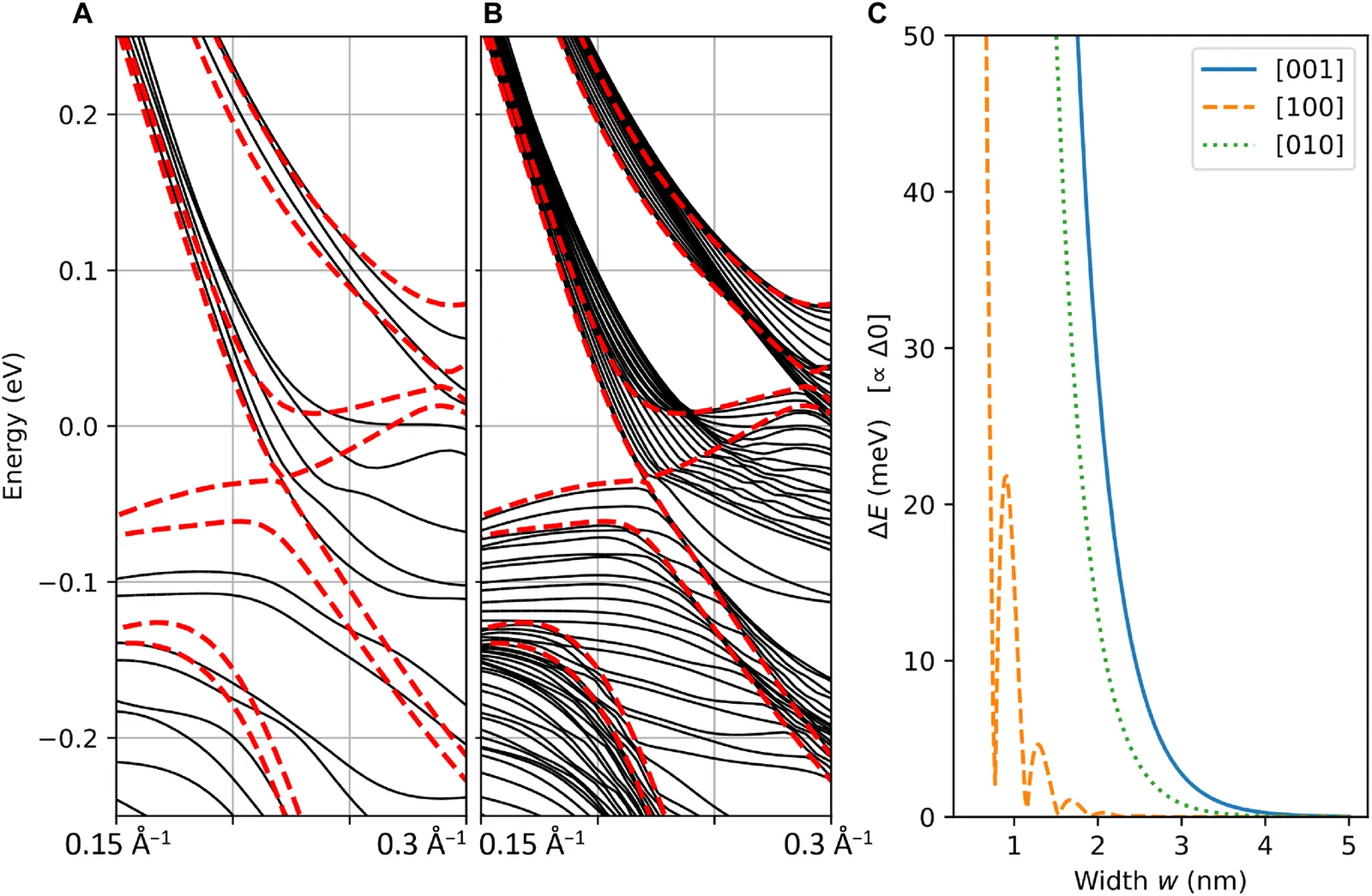
Band structure calculation. (**A**) shows the band structure of the bulk (dashed red lines) around one of the Weyl nodes, superimposed on the band structure for a finite two-layer slab in the [001] direction (black), and (**B**) shows the bulk band structure superimposed on the 10-slab finite band structure. (**C**) shows the continuum model, with parameters fitted to three finite calculations (number of layers being 2, 3, and 5, corresponding to 2.8, 4.2, and 7 nm).

The Weyl points in Td WTe_2_ are located in the ***k***_***x***_ ***k***_***y***_ plane. The coordinates in Cartesian coordinates as well as the Chern numbers are listed in table S3, matching well to published results (*18*). As an upper bound to the decay length of the surface/interface state, we investigate confinement in the *z* direction (as imposed by the sample thickness). In Fig. 5 (A and B), we show the bulk band structure along a path in reciprocal space, parallel to the Γ − *X* direction, but offset in the *y* direction to ensure that the Weyl node (node 4 in table S3) lies on the path. Figure 5A shows a two–unit cell (2.8 nm) cell, indicating a substantial gap and no Weyl node beyond the thermal energy scale of ∼25 meV (corresponding to room temperature). At 10-layer thickness (14 nm), the crossing has acquired bulk-like properties. Figure 5C shows the result of the continuum model, for three different directions. Because experimental domains are hundreds of nanometers wide and tens of nanometers thick, the hybridization gap can be considered zero for all widths *w* regardless of orientation of domain or cut.

Structured optical fields offer a transient and programmable route to define lattice distortions in space and time with nanometer spatial and picosecond temporal resolutions. The interference patterns demonstrated here can be shaped into more complex geometries using multiple beams, enabling deterministic control over size, symmetry, and dimensionality (*24*). Pattern periodicity can be tuned via the wavelength and interference angles, reaching nanometer-scale precision (*15*). Such spatially patterned structural phases enable tailored distributions of electronic and optical properties within a single material. For instance, grating-patterned crystallographic phases can impose a spatial modulation of nonlinear optical susceptibility. As illustrated in fig. S6, second-harmonic generation from these transient grating symmetries produces emission angles that are directly tunable through the grating periodicity.

More broadly, the optical approach demonstrated here introduces a pathway toward programmable topology written by light, where topological phases can be dynamically created, erased, and reconfigured on ultrafast timescales. The resulting topological-trivial heterostructures host interfaces that may support unique boundary electronic states, providing a versatile platform for exploring interface physics and developing nanoscale electronic functionalities. By enabling spatially addressable quantum states with ultrafast control, this strategy establishes a foundation for reconfigurable quantum materials and optically addressable electronic and photonic devices.

In conclusion, we have demonstrated transient phase patterning of the Weyl semimetal Td-WTe_2_ and spatially selective excitation and detection of optical phonons. Using TOGs, we drive a structural transition in nanoscale regions of WTe_2_, resulting in a Td/1T* phase heterostructure. The phase front at the interface between the 1T* and Td domains propagates at a velocity comparable to that of the shear strain wave in the material, revealing a nonlocal phase excitation mediated by the interplay between electronic excitation and lattice strain. Furthermore, the A_1_ optical phonon mode is confined to nanoscale regions aligned with the optical grating, demonstrating precise spatial control of coherent lattice dynamics. We demonstrate that red shifts in the local phonon frequency can be resolved spatially. Tight-binding simulations confirm the robustness of the nano-patterned topological structure. U-STEM provides multidimensional observations of the transient structural states and can be used to pinpoint the spatial locations of the two different phases. These results establish a platform for ultrafast, spatially programmable control of structural phases in two-dimensional materials, offering a route toward optically defined functional architectures and reconfigurable quantum materials.

## MATERIALS AND METHODS

### Sample preparation

WTe_2_ samples were prepared by mechanical exfoliation using adhesive tape (*29*) from a bulk single crystal of Td-WTe_2_ (2D Semiconductor, USA). The sample was then transferred to a piece of polydimethylsiloxane and checked under an optical microscope. Desired flakes were aligned and attached onto a 50-nm Si_3_N_4_ TEM grid (Ted Pella) under the optical microscope. A slanted surface of a Si TEM grid is used as a mirror to partially reflect the pump laser beam. The grid is placed on top of the selected sample flake, with its slanted surface oriented toward the pump laser beam. The grid was coated with aluminum and exhibited a reflectivity of approximately 50%. The laser is aligned such that a portion of the beam is reflected off the slanted surface, while the remainder continues along its original path. The reflected and direct beams interfere at the sample plane, generating a TOG. The resulting laser fluence distribution is governed by the amplitude and phase difference between the interfering beams.

### Ultrafast electron microscope setup

The time-resolved experiment was performed in an ultrafast electron microscope (UEM; based on a JEOL JEM 2100) operating at 200 kV. The electrons are detected by a hybrid pixel detector (CheeTah T3, Amsterdam Scientific Instruments). The electron bunches (probe) were generated through photoemission from a guard ring LaB_6_ cathode by 258-nm laser pulse illumination. The sample was excited by a λ = 1030 nm and 300-fs laser pulse (pump) (Tangerine, Amplitude Systemes). The laser was focused to a spot with an FWHM of ∼200 μm at the sample plane. The time delays between the pump and probe pulses were controlled using a motorized delay stage varying the optical path length of the pump beam. The time resolution of this setup is approximately 1 ps (*30*). The difference in arrival time between the direct and reflected beams in the TOG geometry is 0.07 ps, due to the optical path difference (*31*). WTe_2_ samples were excited at a repetition rate of 12 kHz. The experiments were performed at room temperature. In DF imaging, an objective aperture was inserted to selectively capture the diffraction spot (130) or (150) while blocking all other spots. Consequently, the collected DF images include dynamic information only from the lattice plane corresponding to the selected diffraction spot.

### U-STEM

The UEM is set to operate in scanning mode by directing a nearly parallel nano–electron beam across a sample region. A 10-μm-diameter condenser aperture is used to reduce the electron beam size. The spatial position of the beam is controlled by scanning coils. After scanning the designated sample region, the delay stage shifts to the next time delay. At each scanning step, the direct electron detector captures the resulting diffraction patterns. In line scanning, the scanning trace consists of 40 pixels in one dimension with a step size of 116 nm. In 2D scanning, the scanning area is a 20 × 8 pixel region, with a step size of 152 nm.

### Theory

The Weyl points and the associated coordinates listed in table S3 are calculated using WannierTools (*32*). The underlying Wannier functions were produced with Wannier90 (*33*) based on the Vienna Ab Initio Simulation package (*34*, *35*) using input and structural parameters from (*36*).

## Supplementary Materials

### The PDF file includes:

Figs. S1 to S12 Tables S1 to S3 Legend for movie S1 References
