# Supplementary material for: Ultrafast laser-driven topological phase patterning
Source: Sci Adv. 2026 Sep 18;12(38):eaee8907. doi: 10.1126/sciadv.aee8907 (PMC13588181; doi:10.1126/sciadv.aee8907)
Supplement: Supplementary file 1 [file sciadv.aee8907_sm.pdf]

Science Advances
AAAS

# Supplementary Materials for

## Ultrafast laser-driven topological phase patterning

Jianyu Wu *et al.*

Corresponding author: Jonas Weissenrieder, jonas@kth.se

*Sci. Adv.* **12**, eaee8907 (2026)
DOI: 10.1126/sciadv.aee8907

**The PDF file includes:**

Figs. S1 to S12
Tables S1 to S3
Legend for movie S1
References

**Other Supplementary Material for this manuscript includes the following:**

Movie S1

**Transient optical grating**

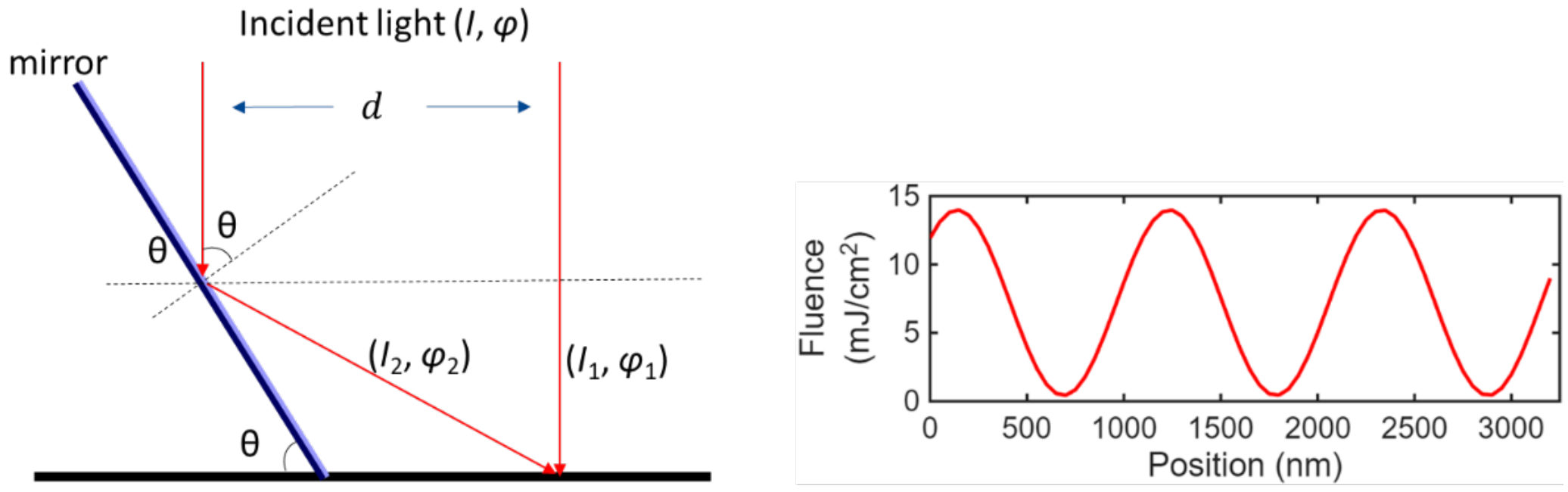


**Figure S1. Calculation of local fluence with transient optical grating.** Left: geometry of laser reflection. Right: local fluence as a function of distance to mirror.

Careful alignment of the pump laser beam to the intersection of the edge of a mirror placed in close proximity to the sample splits the laser beam into two paths. The to the sample direct laser beam and the beam reflected from the slanted side face of the mirror will interfere at the sample plane. The condition for constructive interference determines the spatial period between the interference fringes of the transient optical grating. The incident laser beams are marked as red arrows. The reflecting slanted side face is marked in blue, and the sample plane is marked in black.
The local fluence with two interfering beams is calculated from:

$$F(r) = F1 + F2 + 2\sqrt{F1F2}\cos(\Delta\varphi(r))$$

Where F1 and F2 are the incident fluence of the individual beams. $\Delta\phi(r)$ is the phase difference between the two beams at position $r$ (distance from the edge of the mirror). For the case of Figure 1 in main text, the fluence of the direct incident laser (F1) is 4.8 mJ/cm$^2$, while the reflected laser (F2) is:

$$F2 = F1 * R$$

Where R is the reflectivity of the mirror. Here the R is 0.5 for the Al-coated Si frame. The interfered pump fluence oscillates between 0.41 to 13.69 mJ/cm$^2$ in the TOG with periodicity at approximate 1.1 µm.

**Characterization of sample**

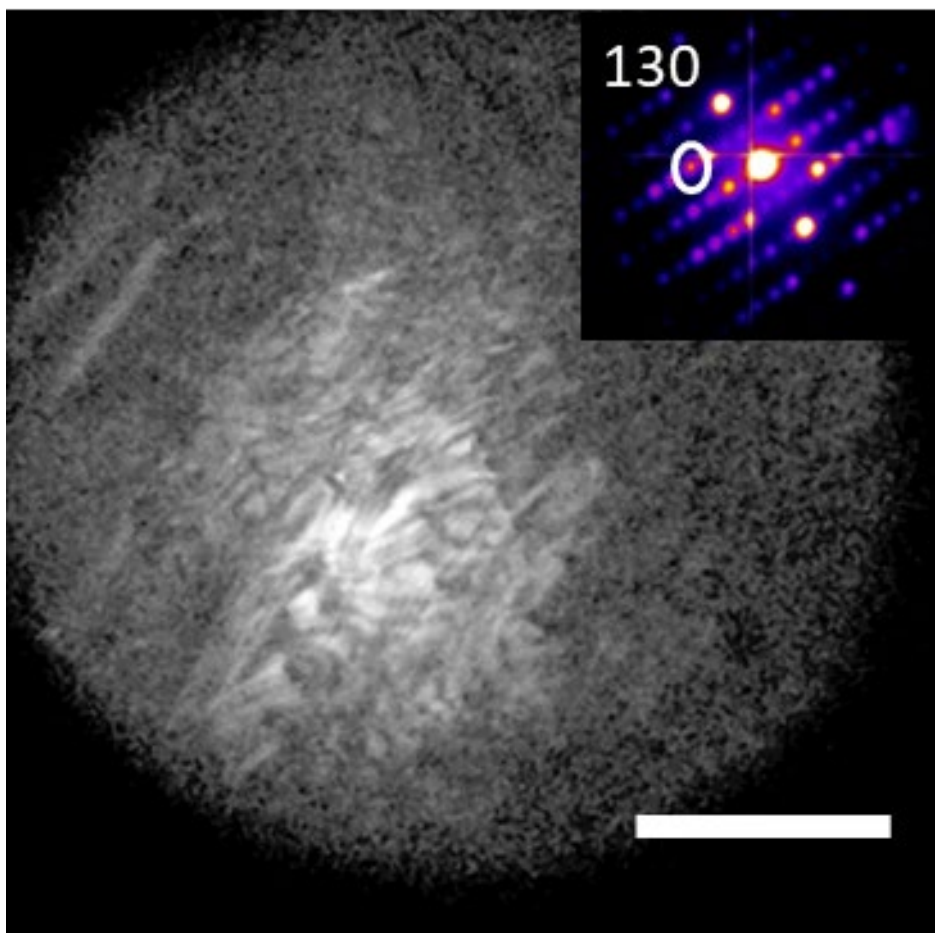


**Figure S2. Dark field UEM.** Dark field image of the sample in Figure 1 of the main text at -2 ps with a diffraction pattern as inset. Scale bar: 2 µm.

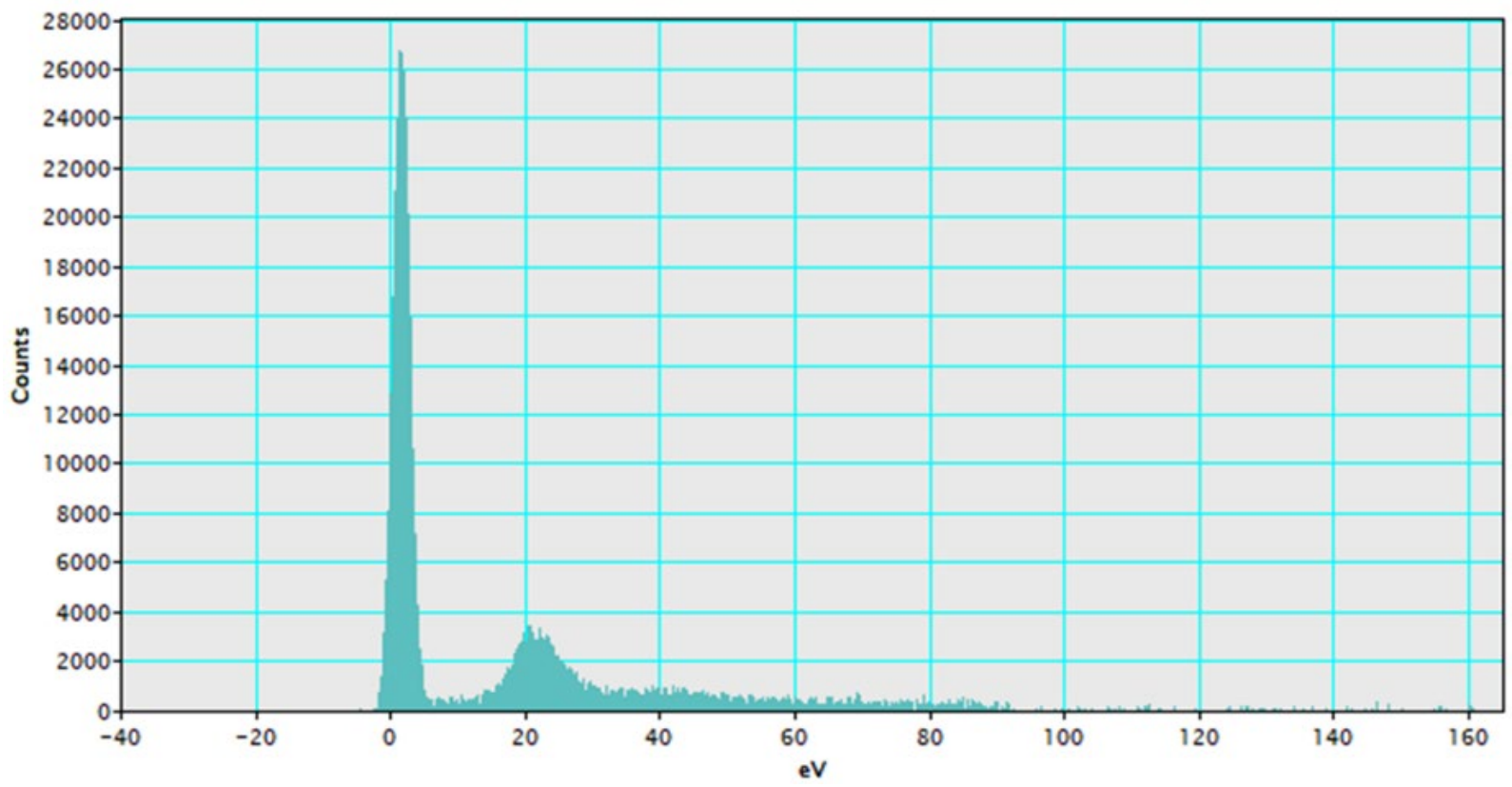


**Figure S3. Electron energy loss spectroscopy**. Electron energy loss spectroscopy (EELS) measured on $WTe_2$ sample.

EELS is used to estimate sample thickness. The thickness at the 0.36 inelastic mean free path (IMFP) is obtained by calculating the log-ratio of the energy loss peak at 20 eV to the 0 eV energy loss peak. The thickness of $Si_3N_4$ background measured from blank area is deducted. With an IMFP of 94 nm for $WTe_2$, (*17*) the thickness is 34 nm.

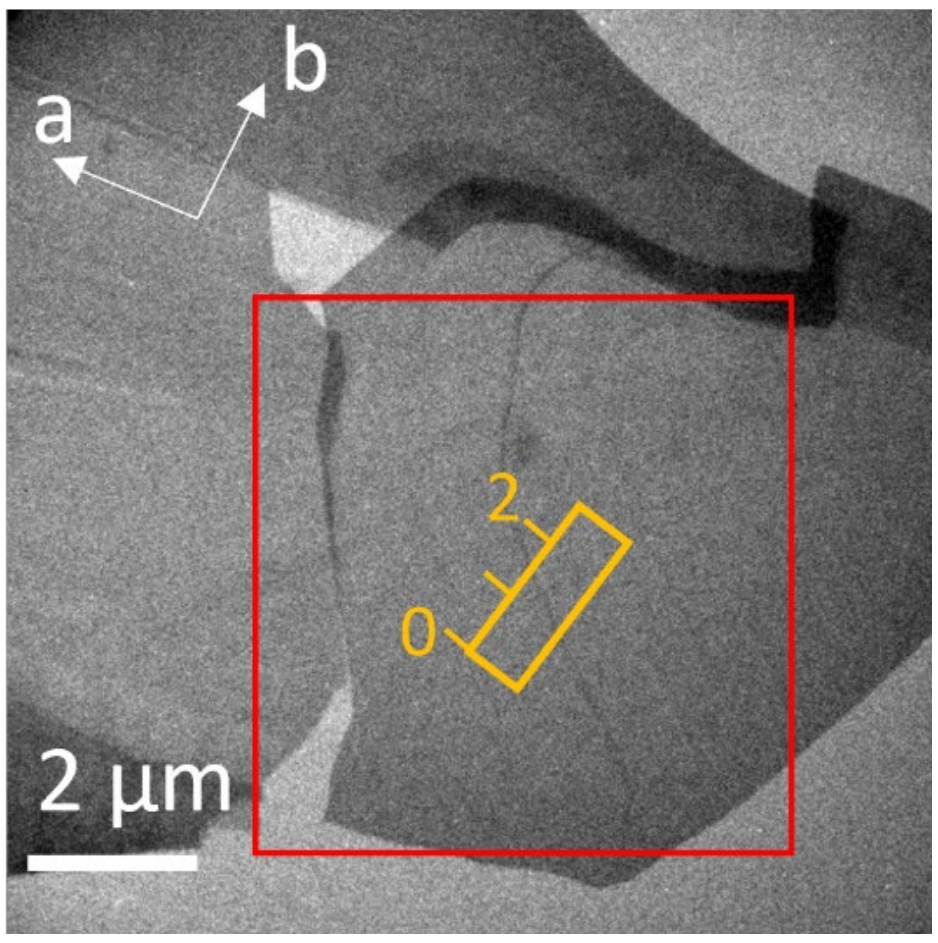


**Figure S4. TEM imaging.** TEM image of the sample in Figure 2 of the main text. The yellow box indicates the region for space-time contour plot in Figure 2(a).

**COMSOL simulation**

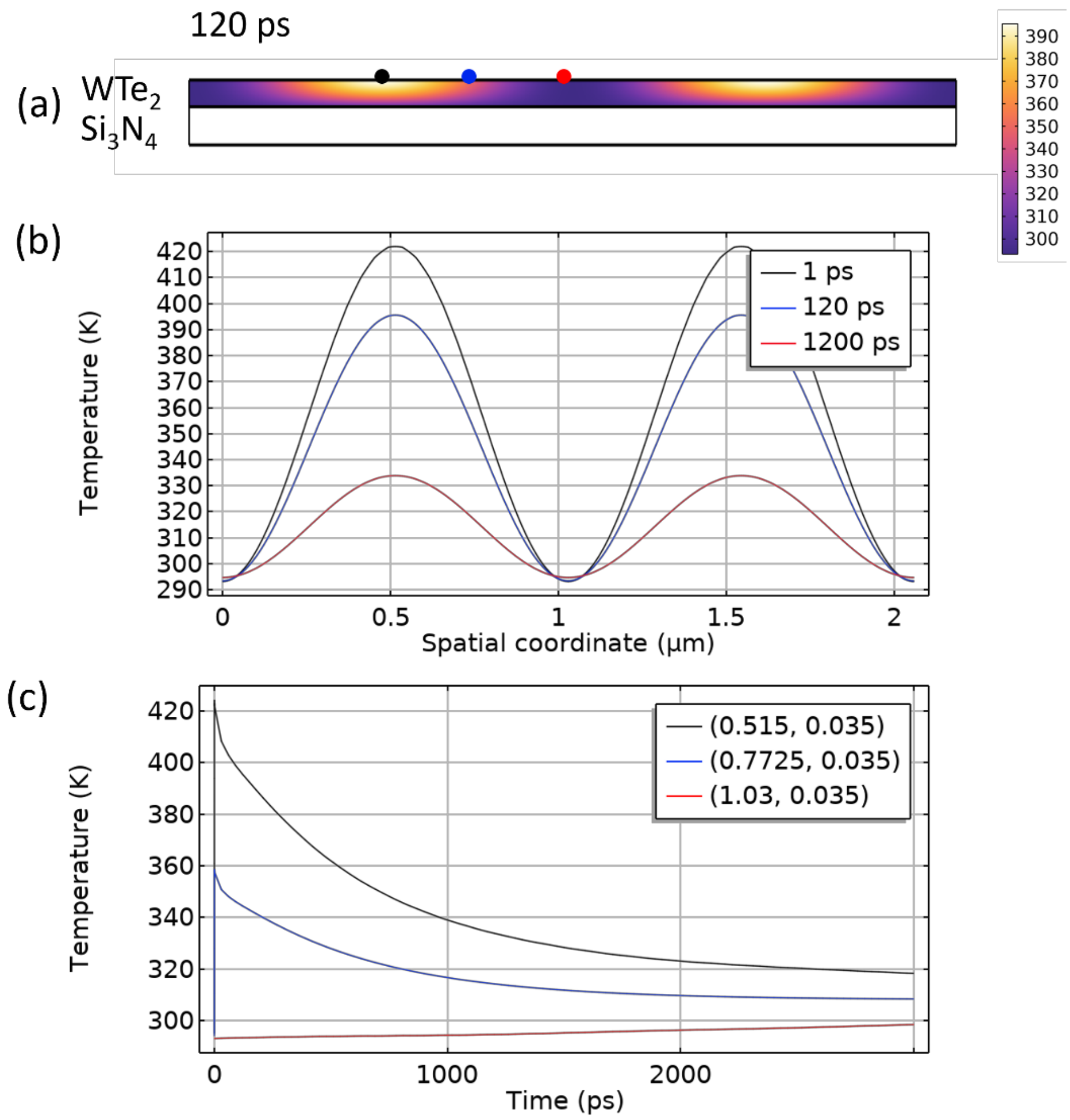


**Figure S5. Finite element simulations.** (a) Model construction in COMSOL Multiphysics. The panel shows the simulated temperature distribution at 120 ps time delay after excitation with a TOG. (b) Temperature profile along the top surface of the model at three different time delays. (c) Simulated temporal temperature evolution at the three

positions indicated by filled circles in (a) in corresponding colors.

COMSOL Multiphysics is employed to simulate the temperature evolution of a $WTe_2$ sample under transient optical grating excitation. The Heat transfer in solid module was utilized for the simulations. The relevant material specific parameters for $WTe_2$ used in the simulation can be found in Table S2. The model comprises a 35 nm $WTe_2$ flake and a 50 nm $Si_3N_4$ substrate with the interface located at z = 0 nm as shown in Figure S5(a). The simulation cell boundaries are set in periodic conditions as for an infinite sample size. The top and bottom boundaries of the sample are left free. Laser-induced lattice heating was simulated by introducing a transient volumetric heat source representing the energy density transferred from the optical pump to the material. The peak power of the heat source was calibrated to correspond to a laser fluence of 1.0 mJ $cm^{-2}$. Its temporal profile followed that of the pump pulse, modelled as a 300 fs pulse starting at time zero. To account for optical attenuation, the heat source amplitude was graded along the sample thickness direction according to the Beer–Lambert law. Laterally, along the in-plane horizontal axis, the heat source intensity was modulated sinusoidally to replicate the interference pattern of the pump beams.

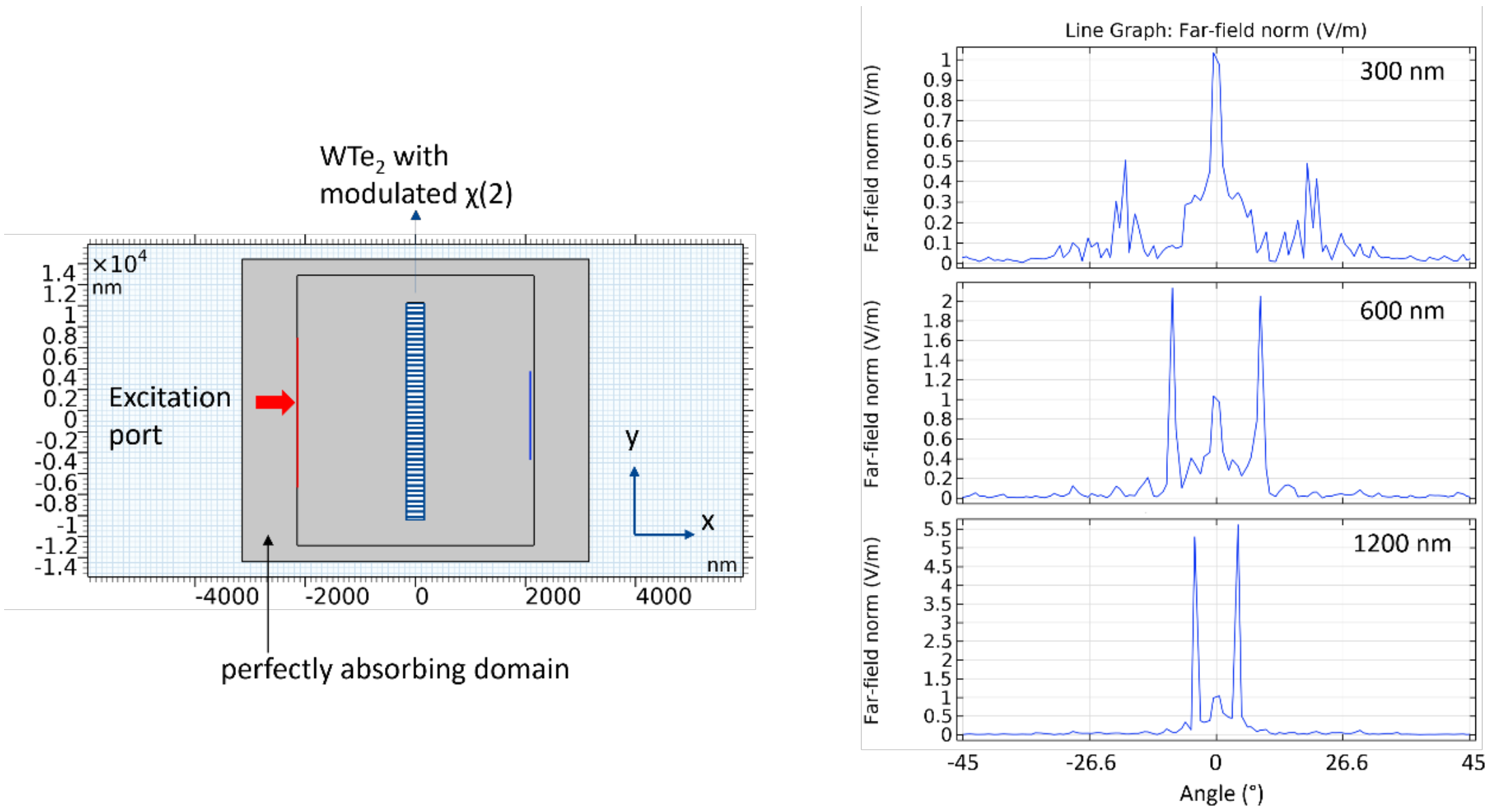


**Figure S6. Second harmonic generation simulated by FEM.** Left: constructed COMSOL model for $WTe_2$ with TOG grating modulated susceptibility. Right: far-field radiation of second harmonics for different grating periodicities.

The model comprises a $WTe_2$ sample of 30 nm thickness (x axis). The nonlinear susceptibility $\chi^{(2)}$ is modulated in gratings (sinusoidal function between Td and 1T*) in the y direction of the model. For Td phase, $\chi_{Td}^{(2)} \approx 95$ pm/V.(*37*) For 1T* phase, $\chi_{1T*}^{(2)} = 0$. The incident wave is polarized in the y-direction and launched at the fundamental frequency *f1* (291 THz) with power at $1\times10^7$ W/m using an excitation port. The second harmonic generation traces (right panel) are for a distance of 2 μm after sample. The boundary of the simulation cell is modeled as a perfectly absorbing layer to represent

an open and nonreflecting infinite volume. The Electromagnetic Waves, Frequency Domain interfaces are defined for the fundamental frequency *f1* and the second harmonic frequency 2**f1*. The two interfaces are coupled using a polarization feature added to each of the interfaces.
For the fundamental interface, the polarization is given by:

$$P_{1y} = 2dE_{2y}E^{*}{}_{2y}$$

and for the second harmonic interface the polarization is given by

$$P_{2y} = dE^{2}{}_{1y}$$

where d is a nonlinear coefficient and calculated by $d=\varepsilon_0 * \chi^{(2)}$, $E_{1y}$ and $E_{2y}$ are the y-component of the electric field at the fundamental frequency and the second harmonic generation. When the spatial periodicity of the grating increases from 300 nm to 1200 nm, the second harmonic far-field radiation shows increasingly sharp lines at the first order interference fringes.

**U-STEM**

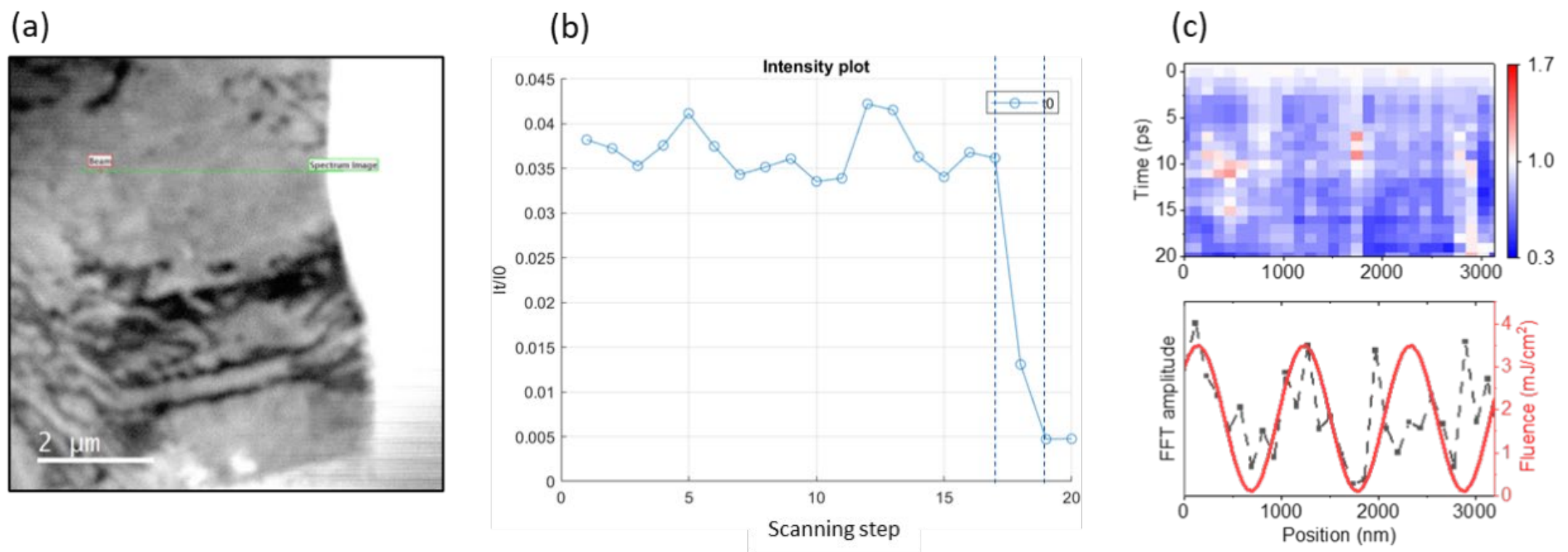


**Figure S7. U-STEM.** U-STEM line scan across a sample edge. (a) STEM image showing the scanned region (green line). (b) Intensity profile of the summed (130) diffraction spot as a function of scanning steps. (c) Upper panel: Dark-field image reconstructed from the (130) reflection. Lower panel: Calculated local pump fluence (red line) and the FFT amplitude at 0.23 THz (dashed line), extracted from the time-domain signal at each spatial column in the upper panel.

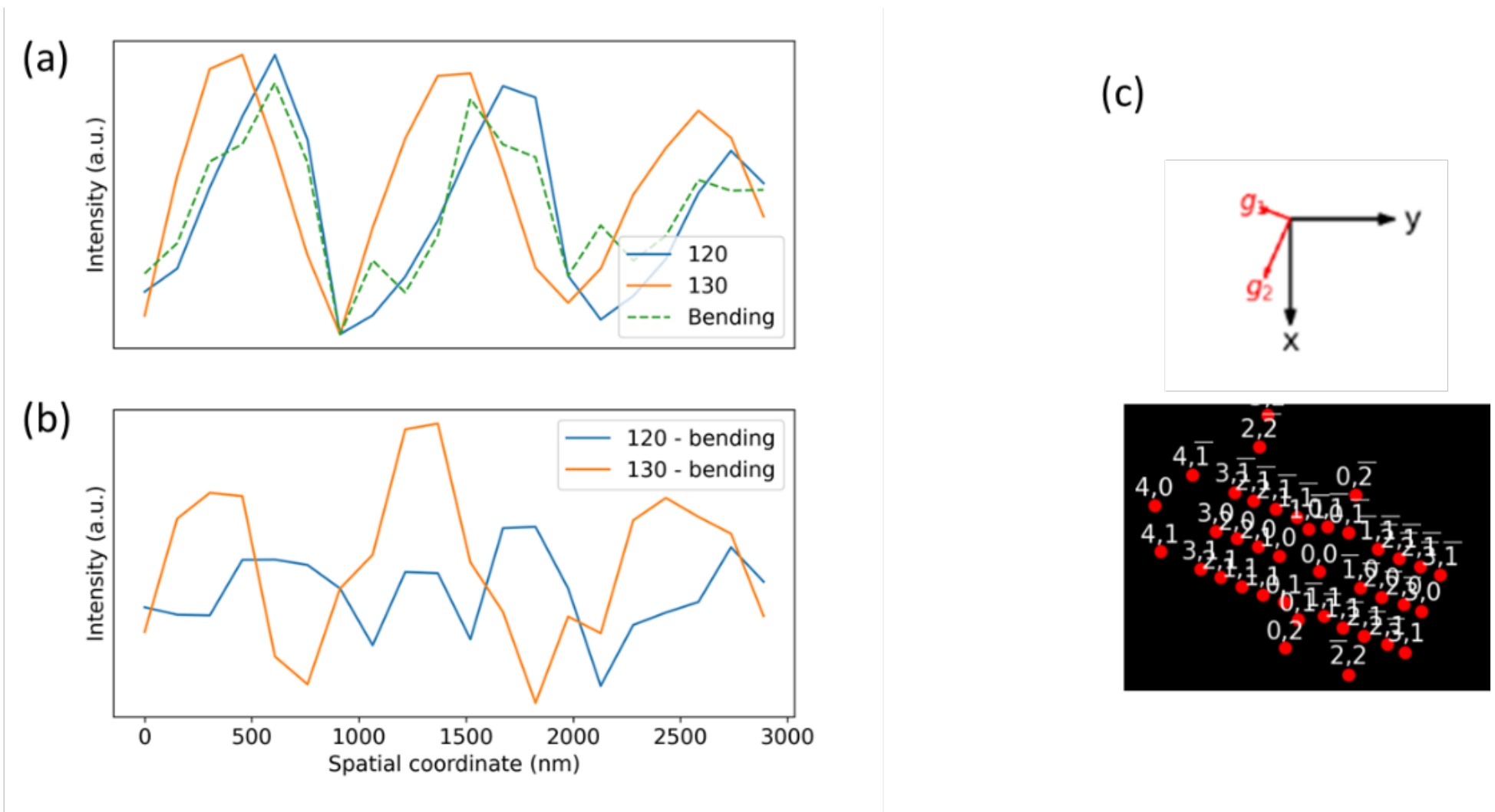


**Figure S8. Local diffraction intensity.** (a) Normalized intensity of the 120 and 130 spots and BF (Bending) from the dataset in Figure 4d of the main text. (b) Intensity traces for the 120 and 130 Bragg spots after removing the contribution from sample bending (BF in a). (c) Bragg spots used for strain calculation, with basis vector g1 and g2 indicated.

The probe beam in U-STEM mode was scanned across the sample edge with a step size of 132 nm. From the 2 steps abrupt intensity drop, the effective probe diameter is determined to be around 200 nm. The images and strain map were calculated using Py4dstem.(*38*) For the reconstructed BF and DF images, intensities were summed along the vertical direction. The BF reflects sample bending and is insensitive to the structural phase transition. The contribution from local sample curvature was therefore obtained by subtracting the BF intensity from its maximum value. This bending component was then removed from the (120) or (130) DF signals, allowing for separating of the structural phase-transition contrast from geometric bending effects.

**Nano beam diffraction**

In nano beam diffraction mode (NBD), the electron beam was converged to a spot with a diameter of 330 nm. The characterization of the electron beam is shown in Figure S9. By scanning the beam across the sample, we find a location for minimal temporal oscillation of the intensity of spot (130) at position 1. This implies a region of low local pump fluence. By shifting the probe beam 330 nm perpendicular to the direction of the TOG fringes, two additional probe positions (2 and 3) are selected to illustrate the evolution of lattice dynamics. The sample position and collected diffraction intensity trace are shown in Figure S10 and Figure S11.

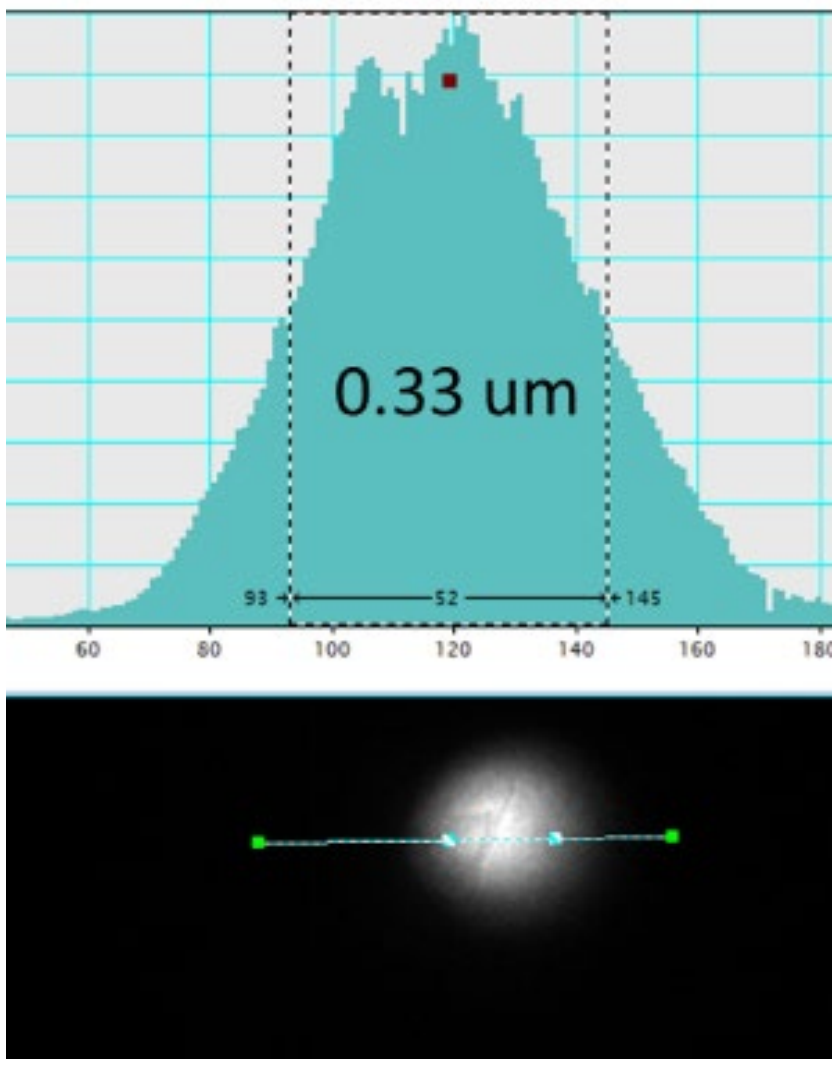


**Figure S9. Characterization of the electron beam in NBD mode.** The full width at half-maximum of the beam is 330 nm.

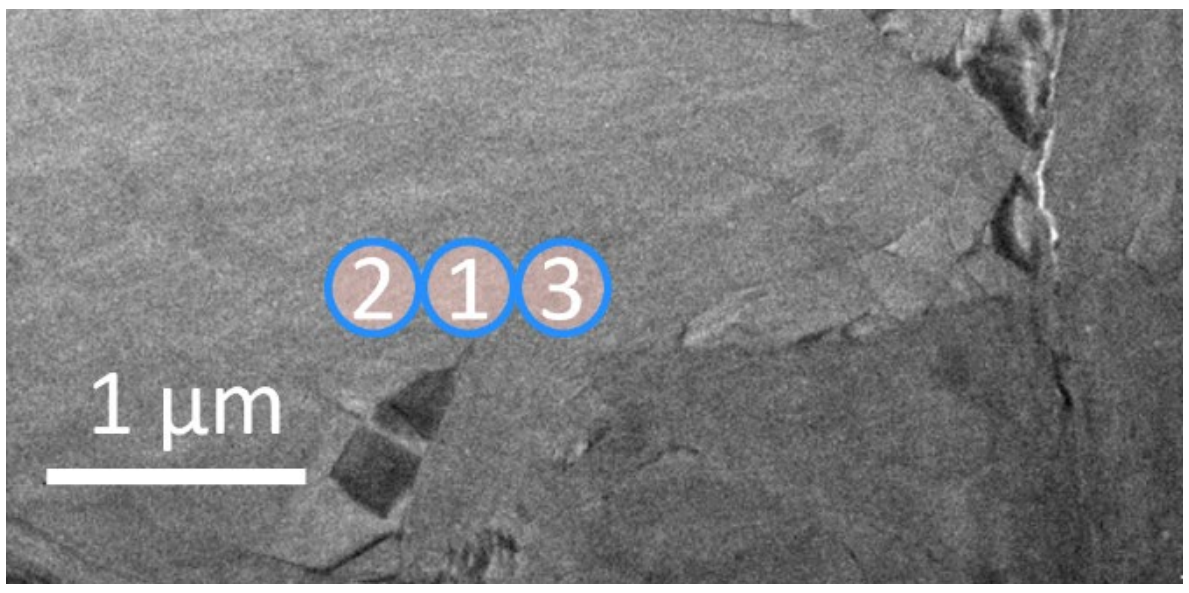


**Figure S10. Sample image and three probe positions in NBD mode.**

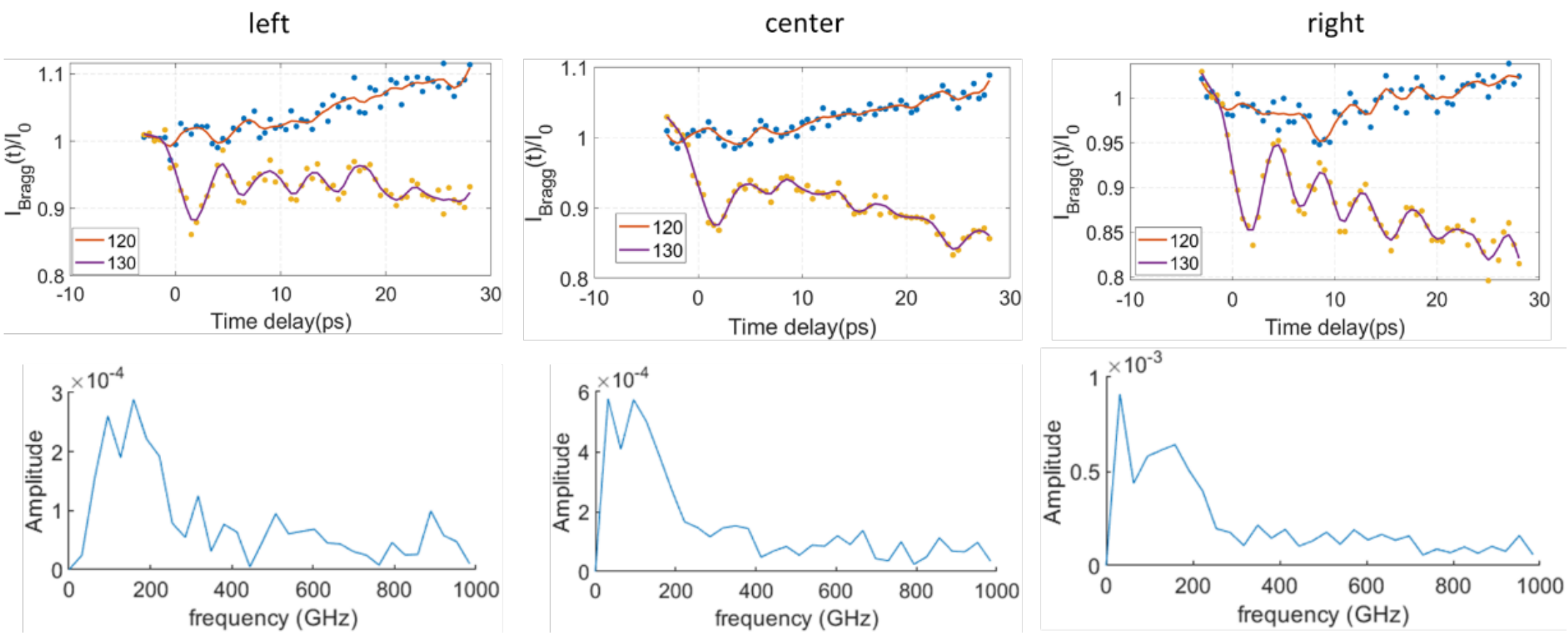


**Figure S11. Local dynamics.** Upper panels: Intensity traces for diffraction spots 120 and 130 at different probe positions. Lower panels: FFT spectrum of spot 130. The results are collected from positions 1 (center), 2 (left) and 3 (right).

## Fitting of interlayer shear phonons

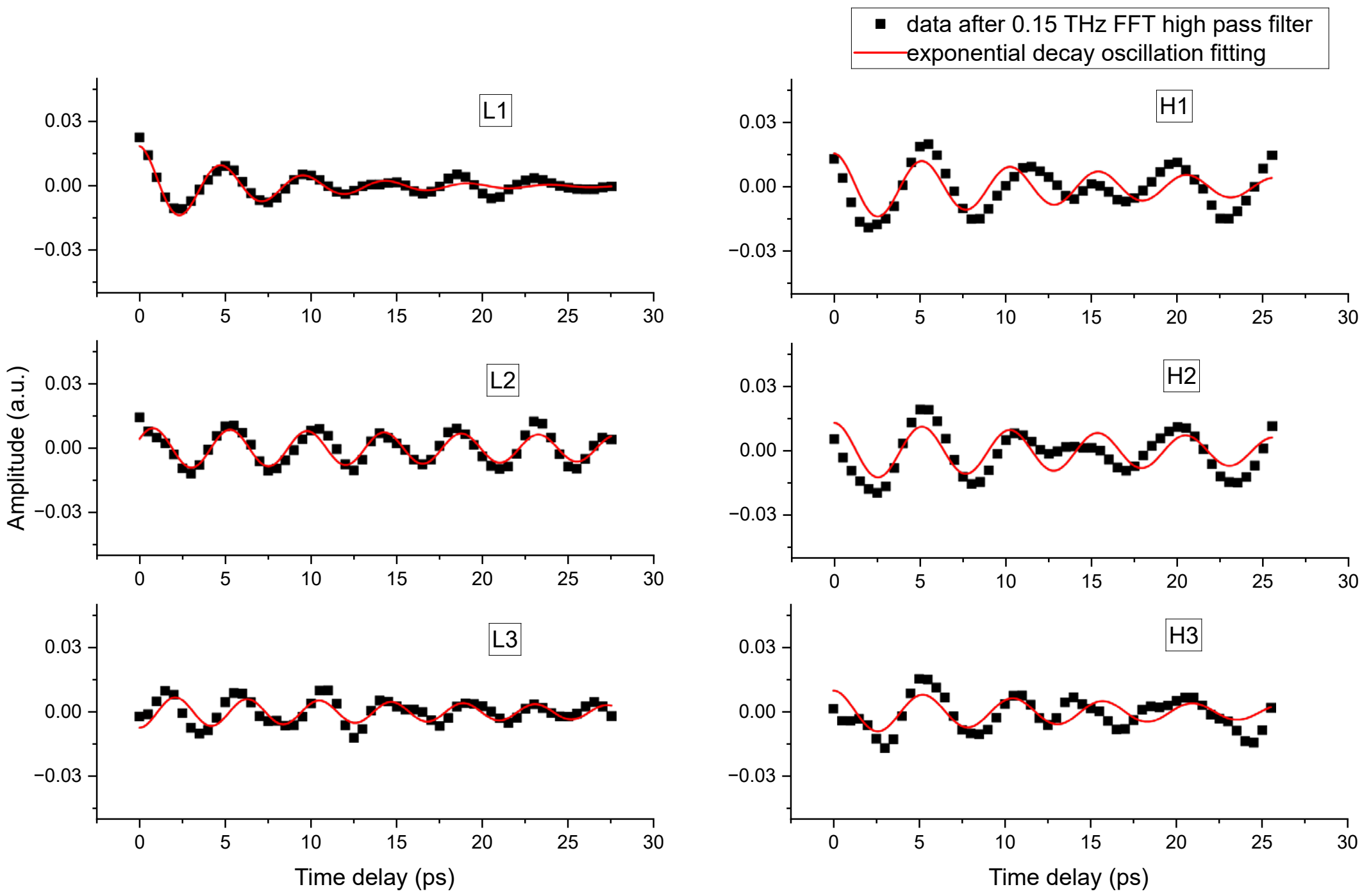


**Figure S12. Fitting of interlayer shear phonons.** The data are extracted from the intensity traces of low excitation regions (L1-L3) and high excitation regions (H1-H3) in Figure 1e of the main text. The red curves represent fits to a damped sinusoidal function.

To isolate the coherent phonon contribution, the raw data were processed using a high-pass Fourier filter with a cutoff frequency of 0.15 THz, removing the slowly varying background arising from the Debye–Waller effect and the structural phase transition. The filtered signals were then fitted using an exponentially damped sinusoidal function:

$$y(x) = A * \exp\left(-\frac{x}{t_1}\right)\cos\left(\frac{2\pi x}{T}\right)$$

where $A$ is the phonon amplitude, $T$ is the oscillation period, $t_1$ is the damping time constant.

The extracted values of $t_1$ and $T$ for each region are summarized in Table S1. The systematically increased oscillation period observed in the high-excitation regions indicates a clear redshift (softening) of the interlayer shear phonon under stronger excitation conditions.

Table S1. Damping constant ($t_1$) and oscillation period ($T$) of the shear phonon obtained from fits to the intensity traces.

| Label | L1 | L2 | L3 | **Average** | H1 | H2 | H3 | **Average** |
|---|---|---|---|---|---|---|---|---|
| $t_1$ (ps) | 7.27 | 59.24 | 34.05 | **33.52** | 19.97 | 35.04 | 23.03 | **26.01** |
| T (ps) | 4.80 | 4.50 | 4.20 | **4.50** | 5.14 | 5.12 | 5.24 | **5.17** |

Table S2. Material properties for $WTe_2$ used in COMSOL simulations.

| **Property** | **Value** | **Unit** | **Reference** |
|---|---|---|---|
| Density | 9863 | kg/m³ | (*39*) |
| Thermal conductivity | k11=1.7,<br>k12=1.6,<br>k13=1.0 | W/(m·K) | (*40*) |
| Heat capacity at constant pressure | 170 | J/(kg·K) | (*41*) |
| Absorption coefficient<br>(at 1030 nm) | 2.59E7 | 1/m | (*42*) |

**Topological Robustness calculation**

Table S3. The coordinates in cartesian coordinates as well as the Chern numbers from tight-binding calculations.

| ID | Kx | ky | kz | C |
|---|---|---|---|---|
| 1 | -0.2195 | -0.0451 | 0.0 | +1 |
| 2 | -0.2202 | -0.0389 | 0.0 | +1 |
| 3 | 0.2195 | 0.0451 | 0.0 | +1 |
| 4 | 0.2202 | 0.0389 | 0.0 | +1 |
| 5 | -0.2194 | 0.0456 | 0.0 | -1 |
| 6 | -0.2202 | 0.0387 | 0.0 | -1 |
| 7 | 0.2194 | -0.0456 | 0.0 | -1 |
| 8 | 0.2202 | -0.0387 | 0.0 | -1 |

*Derivation of continuum model for hybridization gap*

Consider a slab of a Weyl semimetal of thickness $w$ with surfaces at $z = \pm w/2$. The starting point is the low-energy Hamiltonian used in Ref. (*27*) [ Eq.(1)]:

$$H_\chi(\boldsymbol{k}) = \epsilon_0(\boldsymbol{k})\,\mathbb{1} + M(\boldsymbol{k})\sigma_z + v_x\chi\sigma_x k_x + v_y\chi\sigma_y + v_z\sigma_z(k_z - \chi k_0)$$

with $\chi = \pm 1$ the node chirality. Here $\epsilon_0$ and $M$ are even in $\boldsymbol{k}$, including tilt terms for type-II WSMs. In Ref. (*27*), a specific $c_i, m_i$ parametrization of Eq.(1) is used, our derivation holds for the general anisotropic, tilted form.

Based on this Hamiltonian, we now derive a compact formula for the finite-thickness hybridization gap $\Delta E(\boldsymbol{k}_\parallel)$ of two counterface surface states, as developing at an interface, including its exponential decay with $w$ and the tilt-induced oscillatory envelope. For a single surface, we seek states decaying along the slab normal, using $k_z \to -i\partial_z$. Following the ansatz of Eq.(4) in Ref. (*27*):

$$\psi_{\boldsymbol{k}_\parallel z} = \sum_j A_j\, e^{-\lambda_j z}\eta_j(\lambda_j)$$

where the decay parameters $\lambda_j$ are obtained by substituting into the eigenproblem defined in Eqs.(5)-(6) in Ref. (*27*). For type-II WSMs, the roots $\lambda$ generically have nonzero real and imaginary parts, giving oscillatory leakage into the bulk.

Under the assumption that we have a dominant decay constant $\kappa(\boldsymbol{k}_\parallel) = \min_j Re[\lambda_j]$ and oscillation wave number $\alpha(\boldsymbol{k}_\parallel) = Im[\lambda_j]$, they are read off from the semi-infinite solution. With two surfaces a distance $w$ apart, the general finite-slab boundary problem in Ref. (*27*) (Eqs. (12)-(14) or equivalently Eqs.(A23)-(A25)) yields

the exact dispersion $E_{\pm}(\boldsymbol{k}_{\parallel})$ in terms of $\lambda_{1,2}$.

Near a crossing of the two isolated-edge dispersions, degenerate perturbation theory in the top/bottom subspace defined by $\{\psi_T, \psi_B\}$ subspace gives

$$E_{\pm} \approx \frac{E_T + E_B}{2} \pm \frac{1}{2}\sqrt{(E_T - E_B)^2 + 4|t_{\perp}|^2}$$

Where $t_{\perp}(\boldsymbol{k}_{\parallel}) = \langle \psi_T | H | \psi_B \rangle$.

From the explicit slab solutions, the two surface modes have envelopes

$\psi_T(z) \propto e^{-\kappa(z-\frac{w}{2})} e^{i\alpha(z-\frac{w}{2})}$ and $\psi_B(z) \propto e^{+\kappa(z+\frac{w}{2})} e^{i\alpha\prime(z+\frac{w}{2})}$ in the interior.

Sandwiching $H$ between them, the $z$-integral gives $t_{\perp} \propto \frac{(\eta_T{}^{*} v \eta_B) e^{-\kappa w} \sin\left(\frac{\Delta\alpha w}{2}\right)}{\frac{\Delta\alpha}{2}}$,

where $v$ is the local $z$-coupling operator (e.g. $v_z \sigma_z(-i\partial_z)$) $\Delta\alpha = \alpha - \alpha'$.

This reproduces the exponential gap scaling and oscillatory envelope seen in the analytic gap estimate for type-B states [Eq.(29) in Ref. (*27*). Combining the perturbative splitting with the overlap form of $t_{\perp}$, we obtain

$$\Delta E(\boldsymbol{k}_{\parallel}) \approx 2\Delta_0(\boldsymbol{k}_{\parallel}) e^{-\kappa(\boldsymbol{k}_{\parallel})w} \left|\mathrm{sinc}\left(\frac{\Delta\alpha(\boldsymbol{k}_{\parallel})w}{2}\right)\right|.$$

Where $\kappa$ and $\alpha$ are from the semi-infinite solution [Eqs. (A14)-(A17) in Ref. (*27*), and $\Delta_0$ encodes the short-range spinor coupling. In the limit of large width, $\lambda_{1,2}$ reduces to their isolated-edge values found in [Eqs. (A14)-(A15) in Ref. (*27*), and $\Delta E$ matches the perturbative $2|t_{\perp}|$ form. We now proceed to fit $\Delta_0(\boldsymbol{k}_{\parallel})$, $\kappa(\boldsymbol{k}_{\parallel})$, and $\Delta\alpha(\boldsymbol{k}_{\parallel})$ to explicit calculations based on WannierTools, using thicknesses of 2, 3, and 5 unit cells, equivalent to 2.8, 4.2 and 7 nm thicknesses. Note that the minimum of the hybridization gap is not at a constant $\boldsymbol{k}$, we use the value of the original Weyl-point of the bulk. This rough estimate is motivated by the fact that the gap closes to below the thermal limit of ~25 meV (corresponding to room temperature) even in our explicit calculations for finite slabs of a few nm width. For directions other than $z$, $\boldsymbol{k}_{\parallel}$ is calculated based on the Weyl-node position, but the short range decay $\Delta_0$ is used based on the assumption that the decay is slowest for states terminating at vacuum (as opposed to the comparatively smooth matching conditions at phase boundaries).

**Supplementary Movie**

Movie S1. Dark-field imaging of phase evolution in $WTe_2$ pumped by transient optical gratings.

## REFERENCES

1. C. H. Bao, P. Z. Tang, D. Sun, S. Y. Zhou, Light-induced emergent phenomena in 2D materials and topological materials. *Nat. Rev. Phys.* **4**, 33–48 (2022).

2. M. Han, J.-B. Ji, A. Blech, R. E. Goetz, C. Allison, L. Greenman, C. P. Koch, H. J. Wörner, Attosecond control and measurement of chiral photoionization dynamics. *Nature* **645**, 95–100 (2025).

3. A. H. Zewail, Four-dimensional electron microscopy. *Science* **328**, 187–193 (2010).

4. J. G. Horstmann, H. Bockmann, B. Wit, F. Kurtz, G. Storeck, C. Ropers, Coherent control of a surface structural phase transition. *Nature* **583**, 232–236 (2020).

5. P. Tengdin, B. Truc, A. Sapozhnik, L. Kong, N. del Ser, S. Gargiulo, I. Madan, T. Schönenberger, P. R. Baral, P. Che, A. Magrez, D. Grundler, H. M. Rønnow, T. Lagrange, J. Zang, A. Rosch, F. Carbone, Imaging the ultrafast coherent control of a skyrmion crystal. *Phys. Rev. X* **12**, 041030 (2022).

6. M. Sennary, J. Shah, M. Yuan, A. Mahjoub, V. Pervak, N. V. Golubev, M. T. Hassan, Light-induced quantum tunnelling current in graphene. *Nat. Commun.* **16**, 4335 (2025).

7. M. Budden, T. Gebert, M. Buzzi, G. Jotzu, E. Wang, T. Matsuyama, G. Meier, Y. Laplace, D. Pontiroli, M. Riccò, F. Schlawin, D. Jaksch, A. Cavalleri, Evidence for metastable photo-induced superconductivity in $K_3C_{60}$. *Nat. Phys.* **17**, 611–618 (2021).

8. F. Dirnberger, J. Quan, R. Bushati, G. M. Diederich, M. Florian, J. Klein, K. Mosina, Z. Sofer, X. Xu, A. Kamra, F. J. Garcia-Vidal, A. Alu, V. M. Menon, Magneto-optics in a van der Waals magnet tuned by self-hybridized polaritons. *Nature* **620**, 533–537 (2023).

9. E. J. Sie, C. M. Nyby, C. D. Pemmaraju, S. J. Park, X. Shen, J. Yang, M. C. Hoffmann, B. K. Ofori-Okai, R. Li, A. H. Reid, S. Weathersby, E. Mannebach, N. Finney, D. Rhodes, D. Chenet, A. Antony, L. Balicas, J. Hone, T. P. Devereaux, T. F. Heinz, X. Wang, A. M. Lindenberg, An ultrafast symmetry switch in a Weyl semimetal. *Nature* **565**, 61–66 (2019).

10. A. Sood, X. Shen, Y. Shi, S. Kumar, S. J. Park, M. Zajac, Y. Sun, L.-Q. Chen, S. Ramanathan, X. Wang, W. C. Chueh, A. M. Lindenberg, Universal phase dynamics in $VO_2$ switches revealed by ultrafast operando diffraction. *Science* **373**, 352–355 (2021).

11. M. Maiuri, M. Garavelli, G. Cerullo, Ultrafast spectroscopy: State of the art and open challenges. *J. Am. Chem. Soc.* **142**, 3–15 (2020).

12. D. Filippetto, P. Musumeci, R. K. Li, B. J. Siwick, M. R. Otto, M. Centurion, J. P. F. Nunes, Ultrafast electron diffraction: Visualizing dynamic states of matter. *Rev. Mod. Phys.* **94**, 045004 (2022).

13. T. Danz, T. Domröse, C. Ropers, Ultrafast nanoimaging of the order parameter in a structural phase transition. *Science* **371**, 371–374 (2021).

14. G. Cao, S. Jiang, J. Akerman, J. Weissenrieder, Femtosecond laser driven precessing magnetic gratings. *Nanoscale* **13**, 3746–3756 (2021).

15. L. Foglia, R. Mincigrucci, A. A. Maznev, G. Baldi, F. Capotondi, F. Caporaletti, R. Comin, D. De Angelis, R. A. Duncan, D. Fainozzi, G. Kurdi, J. Li, A. Martinelli, C. Masciovecchio, G. Monaco, A. Milloch, K. A. Nelson, C. A. Occhialini, M. Pancaldi, E. Pedersoli, J. S. Pelli-Cresi, A. Simoncig, F. Travasso, B. Wehinger, M. Zanatta, F. Bencivenga, Extreme ultraviolet transient gratings: A tool for nanoscale photoacoustics. *Photoacoustics* **29**, 100453 (2023).

16. F. Bencivenga, R. Mincigrucci, F. Capotondi, L. Foglia, D. Naumenko, A. A. Maznev, E. Pedersoli, A. Simoncig, F. Caporaletti, V. Chiloyan, R. Cucini, F. Dallari, R. A. Duncan, T. D. Frazer, G. Gaio, A. Gessini, L. Giannessi, S. Huberman, H. Kapteyn, J. Knobloch, G. Kurdi, N. Mahne, M. Manfredda, A. Martinelli, M. Murnane, E. Principi, L. Raimondi, S. Spampinati, C. Spezzani, M. Trovò, M. Zangrando, G. Chen, G. Monaco, K. A. Nelson, C. Masciovecchio, Nanoscale transient gratings excited and probed by extreme ultraviolet femtosecond pulses. *Sci. Adv.* **5**, eaaw5805 (2019).

17. S. Ji, O. Grånäs, J. Weissenrieder, Manipulation of stacking order in Td-$WTe_2$ by ultrafast optical excitation. *ACS Nano* **15**, 8826–8835 (2021).

18. A. A. Soluyanov, D. Gresch, Z. Wang, Q. Wu, M. Troyer, X. Dai, B. A. Bernevig, Type-II Weyl semimetals. *Nature* **527**, 495–498 (2015).

19. M. X. Guan, E. Wang, P. W. You, J. T. Sun, S. Meng, Manipulating Weyl quasiparticles by orbital-selective photoexcitation in $WTe_2$. *Nat. Commun.* **12**, 1885 (2021).

20. J. Wu, A. K. Prasad, A. Balatsky, J. Weissenriede, Spatiotemporal determination of photoinduced strain in a Weyl semimetal. *Struct. Dyn.* **11**, 054301 (2024).

21. O. Delaire, K. Marty, M. B. Stone, P. R. C. Kent, M. S. Lucas, D. L. Abernathy, D. Mandrus, B. C. Sales, Phonon softening and metallization of a narrow-gap semiconductor by thermal disorder. *Proc. Natl. Acad. Sci. U.S.A.* **108**, 4725–4730 (2011).

22. B. He, C. Zhang, W. Zhu, Y. Li, S. Liu, X. Zhu, X. Wu, X. Wang, H.-h. Wen, M. Xiao, Coherent optical phonon oscillation and possible electronic softening in $WTe_2$ crystals. *Sci. Rep.* **6**, 30487 (2016).

23. P. D. Nellist, Chapter 2: The Principles of STEM Imaging, in *Scanning Transmission Electron Microscopy: Imaging and Analysis*, S. J. Pennycook, P. D. Nellist, Eds. (Springer New York, 2011), pp. 91–115.

24. J. Wu, G. Cao, Y. Fan, S. P. Dash, D. Yu, J. Weissenrieder, Optically driven formation of tailored phonon cavities. *Adv. Sci.* **13**, e14963 (2026).

25. C. Ophus, Four-dimensional scanning transmission electron microscopy (4D-STEM): From scanning nanodiffraction to ptychography and beyond. *Microsc. Microanal.* **25**, 563–582 (2019).

26. F. S. Yasin, J. Masell, M. Birch, K. Karube, Y. Taguchi, T.-H. Arima, Y. Tokura, X. Yu, A. R. Lupini, Development of Lorentz four-dimensional scanning transmission electron microscopy for quantitative measurements of in-situ current-driven magnetic domain wall deformations. *Microsc. Microanal.* **31**, ozaf048.891 (2025).

27. E. Benito-Matías, R. A. Molina, Surface states in topological semimetal slab geometries. *Phys. Rev. B* **99**, 075304 (2019).

28. M. H. Berntsen, O. Götberg, B. M. Wojek, O. Tjernberg, Direct observation of decoupled Dirac states at the interface between topological and normal insulators. *Phys. Rev. B* **88**, 195132 (2013).

29. K. S. Novoselov, A. K. Geim, S. V. Morozov, D. Jiang, Y. Zhang, S. V. Dubonos, I. V. Grigorieva, A. A. Firsov, Electric field effect in atomically thin carbon films. *Science* **306**, 666–669 (2004).

30. S. Ji, L. Piazza, G. Cao, S. T. Park, B. W. Reed, D. J. Masiel, J. Weissenrieder, Influence of cathode geometry on electron dynamics in an ultrafast electron microscope. *Struct. Dyn.* **4**, 054303 (2017).

31. Y. Fan, G. Cao, S. Jiang, J. Åkerman, J. Weissenrieder, Spatiotemporal observation of surface plasmon polariton mediated ultrafast demagnetization. *Nat. Commun.* **16**, 873 (2025).

32. Q. Wu, S. Zhang, H.-F. Song, M. Troyer, A. A. Soluyanov, WannierTools: An open-source software package for novel topological materials. *Comput. Phys. Commun.* **224**, 405–416 (2018).

33. A. A. Mostofi, J. R. Yates, G. Pizzi, Y.-S. Lee, I. Souza, D. Vanderbilt, N. Marzari, An updated version of wannier90: A tool for obtaining maximally-localised Wannier functions. *Comput. Phys. Commun.* **185**, 2309–2310 (2014).

34. G. Kresse, J. Furthmüller, Efficiency of ab-initio total energy calculations for metals and semiconductors using a plane-wave basis set. *Comput. Mater. Sci.* **6**, 15–50 (1996).

35. G. Kresse, D. Joubert, From ultrasoft pseudopotentials to the projector augmented-wave method. *Phys. Rev. B* **59**, 1758–1775 (1999).

36. S. Ji, O. Granas, A. Kumar Prasad, J. Weissenrieder, Influence of strain on an ultrafast phase transition. *Nanoscale* **15**, 304–312 (2022).

37. L. L. Lovely, Optical second harmonic generation in $WTe_2$ (Simon Fraser University, 2020); https://summit.sfu.ca/item/34425.

38. B. H. Savitzky, S. E. Zeltmann, L. A. Hughes, H. G. Brown, S. Zhao, P. M. Pelz, T. C. Pekin, E. S. Barnard, J. Donohue, L. Rangel DaCosta, E. Kennedy, Y. Xie, M. T. Janish, M. M. Schneider, P. Herring, C. Gopal, A. Anapolsky, R. Dhall, K. C. Bustillo, P. Ercius, M. C. Scott, J. Ciston, A. M. Minor, C. Ophus, py4DSTEM: A software package for four-dimensional scanning transmission electron microscopy data analysis. *Microsc. Microanal.* **27**, 712–743 (2021).

39. B. Rahman Rano, I. M. Syed, S. H. Naqib, Elastic, electronic, bonding, and optical properties of $WTe_2$ Weyl semimetal: A comparative investigation with $MoTe_2$ from first principles. *Results Phys.* **19**, 103639 (2020).

40. Y. Chen, B. Peng, C. Cong, J. Shang, L. Wu, W. Yang, J. Zhou, P. Yu, H. Zhang, Y. Wang, C. Zou, J. Zhang, S. Liu, Q. Xiong, H. Shao, Z. Liu, H. Zhang, W. Huang, T. Yu, In-plane anisotropic thermal conductivity of few-layered transition metal dichalcogenide Td-$WTe_2$. *Adv. Mater.* **31**, e1804979 (2019).

41. G. Liu, H. Y. Sun, J. Zhou, Q. F. Li, X.-G. Wan, First-principles study of lattice thermal conductivity of Td–$WTe_2$. *New J. Phys.* **18**, 033017 (2016).

42. M. N. Polyanskiy, Refractiveindex.info database of optical constants. *Sci. Data* **11**, 94 (2024).
